# Facile photosynthesis of novel porphyrin-derived nanocomposites containing Ag, Ag/Au, and Ag/Cu for photobactericidal study

**DOI:** 10.1038/s41598-023-34745-0

**Published:** 2023-05-26

**Authors:** Sajedeh Tehrani Nejad, Rahmatollah Rahimi, Mahboubeh Rabbani, Sadegh Rostamnia

**Affiliations:** 1 Inorganic Group, Department of Chemistry, Iran University of Science and Technology (IUST), Tehran, 16846-13114 Iran; 2 Organic and Nano Group (ONG), Department of Chemistry, Iran University of Science and Technology (IUST), PO Box 16846-13114, Tehran, Iran

**Keywords:** Chemistry, Catalysis, Chemical biology, Inorganic chemistry, Materials chemistry, Medicinal chemistry

## Abstract

In this research, the one-step synthesis of novel porphyrin-based nanocomposites was performed easily using a photochemical under visible light illumination strategy. As a result, the focus of this research is on synthesizing and using decorated ZnTPP (zinc(II)tetrakis(4-phenyl)porphyrin) nanoparticles with Ag, Ag/AgCl/Cu, and Au/Ag/AgCl nanostructures as antibacterial agents. Initially, ZnTPP NPs were synthesized as a result of the self-assembly of ZnTPP. In the next step, in a visible-light irradiation photochemically process, the self-assembled ZnTPP nanoparticles were used to make ZnTPP/Ag NCs, ZnTPP/Ag/AgCl/Cu NCs, and ZnTPP/Au/Ag/AgCl NCs. A study on the antibacterial activity of nanocomposites was carried out for *Escherichia coli*, and *Staphylococcus aureus* as pathogen microorganisms by the plate count method, well diffusion tests, minimum inhibitory concentration (MIC), and minimum bactericidal concentration (MBC) values determination. Thereafter, the reactive oxygen species (ROS) were determined by the flow cytometry method. All the antibacterial tests and the flow cytometry ROS measurements were carried out under LED light and in dark. The (3-(4,5-dimethylthiazol-2-yl)-2,5-diphenyltetrazolium bromide (MTT) assay was applied to investigate the cytotoxicity of the ZnTPP/Ag/AgCl/Cu NCs, against Human foreskin fibroblast (HFF-1) normal cells. Due to the specific properties such as admissible photosensitizing properties of porphyrin, mild reaction conditions, high antibacterial properties in the presence of LED light, crystal structure, and green synthesis, these nanocomposites were recognized as kinds of antibacterial materials that are activated in visible light, got the potential for use in a broad range of medical applications, photodynamic therapy, and water treatment.

## Introduction

In recent years, experimental and industrial achievements in nanotechnology have spawned a new approach in applied sciences, resulting in the growth of interdisciplinary activities in industries, the environment, and medicine^1–3^. Due to the importance of preventing harmful bacterial damage, antibacterial agents are being developed. As a result, nanomaterials are very beneficial treatments regarded due to their particular qualities against bacterial infections caused by misuse of antibiotics, which has led to bacterial resistance and a global threat to human health. Furthermore, the creation of biocompatible antibacterial agents is one of the most pressing topics for scientists^4,5^. *E. coli* is a pernicious pathogen and a gram-negative bacillus. As well, the eradication of *E. coli* is becoming increasingly challenging. Humans get diarrhea from *Staphylococcus aureus* (Gram-positive) and *Escherichia coli* (Gram-negative) bacteria after drinking contaminated water. As a result, a healthy drinking water supply is critical to human health^6,7^.

The porphin core's sturdy macrocyclic structure makes it a good anchoring point for metal atom complexation^8,9^. The studies of porphyrins synthesis, structure, assemblies, and applications have always intrigued the scientific community^10^. Porphyrins are species of supramolecules and have a wide range of photophysical and photochemical properties, high photosensitizing efficiencies, superior energy, electron transfer capacities, and excellent light-harvesting potential including strong light absorption in the visible region while their energy levels can be easily adjusted to match those of donor materials using a suitable molecular design^11,12^. Porphyrins are also widely utilized in antimicrobial photodynamic therapy^13–15^.

Free radicals, or more likely singlet oxygen, can be generated by the light-exposed porphyrin. This process is dependent on the type of porphyrin as a photosensitizer and light source employed. These species are extremely reactive and can interact with nearly every cell component, including protein, lipid, and nucleic acid. Some reactive by-products, such as reactive oxygen species, can be produced as a result of this interaction (ROS). These species can cause further harm and cell death^16^.

**Figure 1:**
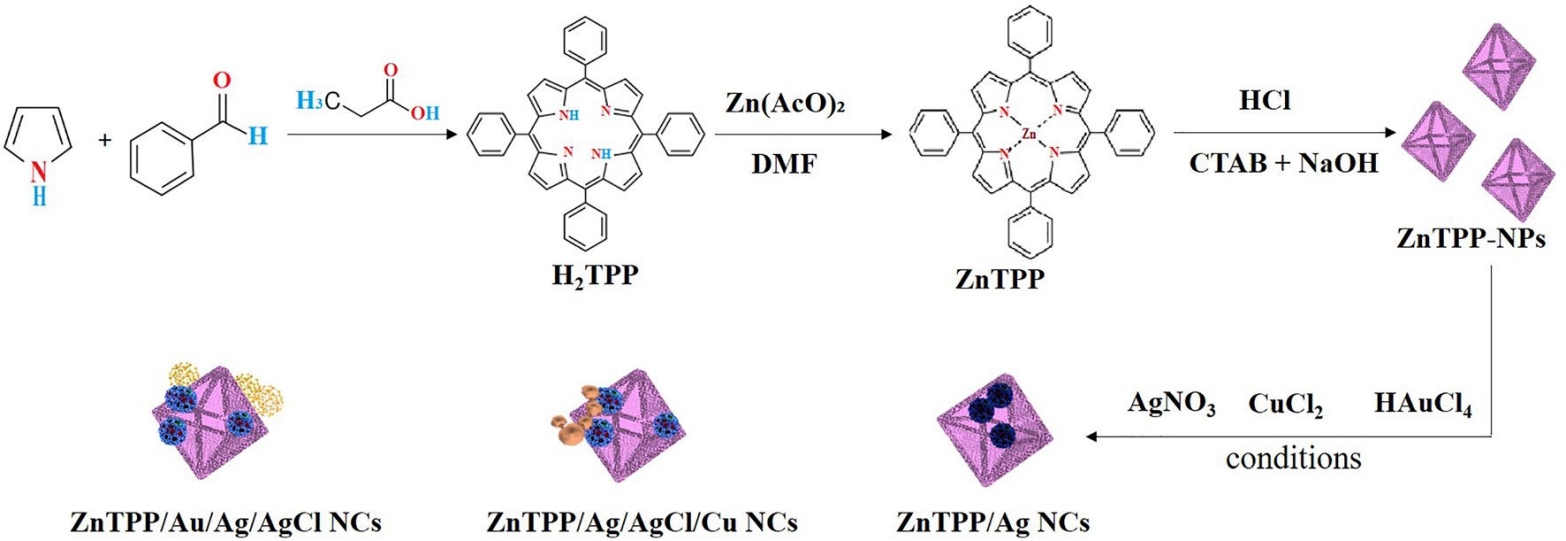
Schematic preparation of ZnTPP/Ag NCs, ZnTPP/Ag/AgCl/Cu NCs, and ZnTPP/Au/Ag/AgCl NCs.

Metallic nanoparticles, particularly silver (Ag) nanoparticles, have been demonstrated to be potent antibacterial agents with a wide range of antibacterial activity^17,18^. Nanomaterials including copper, are of tremendous interest because of their widespread availability, inexpensive cost, and similarities to noble metal characteristics. As well it can also be used as a bactericidal and antimicrobial agent to coat hospital equipment^19,20^. Gold nanoparticles (Au NPs) are emerging as ideal candidates because of their significant biomedical efficacy^21,22^. By combining materials with potential properties, new materials with better and more effective performance can be created. In this work, we synthesized multi-component nanocomposites containing ZnTPP nanostructures and several metal components in the form of ZnTPP/Ag NCs, ZnTPP/Ag/AgCl/Cu NCs, and ZnTPP/Au/Ag/AgCl NCs by using the photochemical method. As well, we proved the effectiveness of these nanoparticles by examining these nanoparticles via LED lamp light on two types of human pathogenic bacteria. In addition, an important advantage of this nanocomposite is the better killing of *E. coli* compared to *S. aureus* bacteria in the presence of light can be mentioned for photodynamic therapy studies. Moreover, the antibacterial potential of synthesized nanocomposites was also studied using the MIC method against the most common drug-resistant microorganisms in the medical field which was examined in dark and LED light in the presence of nanocomposites. The synergistic effect of these metals was investigated in the available approach and recommended as a workable method for use in removing bacterial contaminants from drinking water and medicinal properties (Fig. 1).

## Experimental

### Materials and methods

Each of the utilized chemicals in this work was of analytical grade agents. Silver nitrate (AgNO_3_), Tetra chloroauric (III) acid trihydrate (HAuCl_4_·3H_2_O), Copper chloride dihydrate (CuCl_2_·2H_2_O), sodium hydroxide (NaOH), hydrochloric acid (HCl 37%), methylene blue (MB), Ethanol 96%, pyrrole (CH_4_NCH_3_) and propionic acid (C_3_H_6_O_2_) were prepared from Merck Company. For porphyrin metalation, zinc acetate dihydrate (Zn(CH_3_CO_2_)_2_·2H_2_O) and dimethylformamide (DMF) were purchased from Merck Company as well. Cetyltrimethylammonium bromide (CTAB), l-ascorbic acid (AA), and polyacrylic acid (PAA) were prepared from Sigma-Aldrich Company. For the preparation of the solutions, deionized water was used. All of the reagents were applied with no further purification except pyrrole, which was first distilled and utilized for porphyrin synthesis.

Powder X-ray diffraction (PXRD) analysis was performed on a D Jeoljdx-8030 X-ray powder diffractometer with Cu Kα (l = 0.154 nm) radiation (40 kV, 30 mA). For morphological investigation of the nanocomposites and elemental analysis, EDS Semi-quantitative, and map scanning analysis, the MIRA3 TESCAN—XMU Field emission electron microscope (FE-SEM) was used. By using a transmission electron microscope (model: EM 208S) AT 100 kV, the nanoparticles were determined. For photocatalytic synthesis, the incandescent Halogen lamp (DONAR DN-30059, 82 V, 360 W). The Fourier transform infrared (FT-IR) analyses were utilized on a Shimadzu FTIR-8400S spectrophotometer using a KBr pellet for sample preparation. To detect the porphyrin’s structure, a double-beam UV–visible spectrometer (Shimadzu UV-1700) at room temperature in the range of 400–700 nm was used.

### Preparation of Zinc meso-tetraphenyl porphyrin (ZnTPP)

The tetraphenyl porphyrin (H_2_TPP) was synthesized according to the procedure of Adler^23^, firstly the pyrrole was distilled. Then, 9 mmol from each distilled pyrrole and benzaldehyde were refluxed for 4 h in 170 mL of propionic acid. The purification of the product was carried out by chromatographic column. From there on for preparing ZnTPP, 1 mmol of prepared H_2_TPP and 2 mmol of Zn(Ac)_2_ were refluxed in 70 mL DMF (155 °C) for 6 h^24^.

### Preparation of ZnTPP NPs

ZnTPP nanoparticles were prepared through the acid–base neutralization self-assembly procedures. In this route, the dispersed solution of 10 mL of ZnTPP (0.1 M) in HCl solution (0.2 M) was injected into 19 mL of stirring aqueous solution of cetrimonium bromide (CTAB), (0.01 M) and NaOH (0.008 M) at STP conditions. The stirring of the mixture was continued for 40 min, then centrifuged at 10,000 rpm, washed with deionized water, and dried.

### Preparation of ZnTPP/Ag NCs

For the synthesis of ZnTPP/Ag NCs, 2 mL of AgNO_3_ solution (50 mM) with 0.5 mL of the solution of l-ascorbic acid (0.1 M) and 10 mL of dispersed ZnTPP nanostructures (0.15 mM) were mixed in a glass vial and stirred for 10 min under a halogen light lamp (360-Watt, 82 Volt). The product was centrifuged at 10,000 rpm for 15 min and then washed with deionized water and dried. The achieved compound, ZnTPP/Ag NCs**,** was named A**.**

### Preparation of ZnTPP/Ag/AgCl/Cu NCs

The ZnTPP/Ag/AgCl/Cu NCs was synthesized through the one-step addition of the 1 mL of AgNO_3_ solution (50 mM), 1 mL of CuCl_2_ solution (50 mM), 0.5 mL of the solution of l-ascorbic acid (0.1 M), and 10 mL of dispersed ZnTPP nanostructures (0.15 mM) into a glass vial. And stirred them under a halogen light lamp (360-Watt, 82 Volt) for 10 min. Washing the sediment with deionized water and drying it led to obtaining the ZnTPP/Ag/AgCl NCs nanocomposite which was named “B”.

### Preparation of ZnTPP/Au/Ag/AgCl NCs

The ZnTPP/Au/Ag/AgCl NCs was synthesized through the one-step addition of the 1 mL of HAuCl_4_·3H_2_O solution (50 mM), 1 mL of AgNO_3_ solution (50 mM), 1 mL of CuCl_2_ solution (50 mM), 0.5 mL of the solution of l-ascorbic acid (0.1 M), and 10 mL of dispersed ZnTPP nanostructures (0.15 mM) into a glass vial under stirring and Halogen lamp for 10 min. Then the sediment was washed with deionized water and dried. This nanocomposite was named “G”.

### Microorganism preparation

For antimicrobial tests, the *Escherichia coli* (ATCC 25922) and *Staphylococcus aureus* (ATCC 25923) strains were purchased from the microorganism bank of the Scientific Industrial Research Organization of Iran.

### Cell line for cytotoxicity assay

The human foreskin fibroblast (HFF-1) cell line was bought from the national cell bank of Iran (Pasture Institute, Tehran, Iran).

### Statistical analysis

Statistical analysis was carried out utilizing package SPSS v16.0 software.

## Results and discussion

In this research and in the continuation of our recent research in nanotechnology^25–27^, mono and bimetallic nanocomposites were synthesized by a simple strategy with an eco-friendly and green solvent. In the synthesis of bi-metallic porphyrin-based nanocomposites, we utilized the values of the salt consumption ratio as follows (Fig. 1): 100% of AgNO_3_, (50 mM) was used as a salt solution in the synthesis of the “A” nanocomposite. 50% of AgNO_3_ with 50% of CuCl_2_·2H_2_O was applied in the synthesis of the “B” nanocomposite. By utilizing 33% of AgNO_3_, and 33% of CuCl_2_·2H_2_O, and 33% of HAuCl_4_*·*3H_2_O salts in the synthesis of the nanocomposite, we gained the compound which was attributed to the ZnTPP/Au/Ag/AgCl NCs (“G” nanocomposite).

The synthesized nanocomposite properties were studied identification in terms of the EDX spectrum, mapping analysis, UV–Vis spectroscopy, FE-SEM images, PXRD pattern, FT-IR analysis, and TEM images. Furthermore, antibacterial properties are studied by an agar disk diffusion, MIC, MBC, and colony counter method in the presence of nanocomposite with LED light and dark. The role of intracellular reactive oxygen species (ROS) in the antibacterial mechanism of the ZnTPP/Ag/AgCl/Cu NCs, the nanocomposite with the best antibacterial result, was studied by flow cytometry under LED light and dark as well. The cytotoxicity of ZnTPP/Ag/AgCl/Cu NCs was evaluated on human foreskin fibroblasts (HFF-1) cells by MTT test.

**Figure 2:**
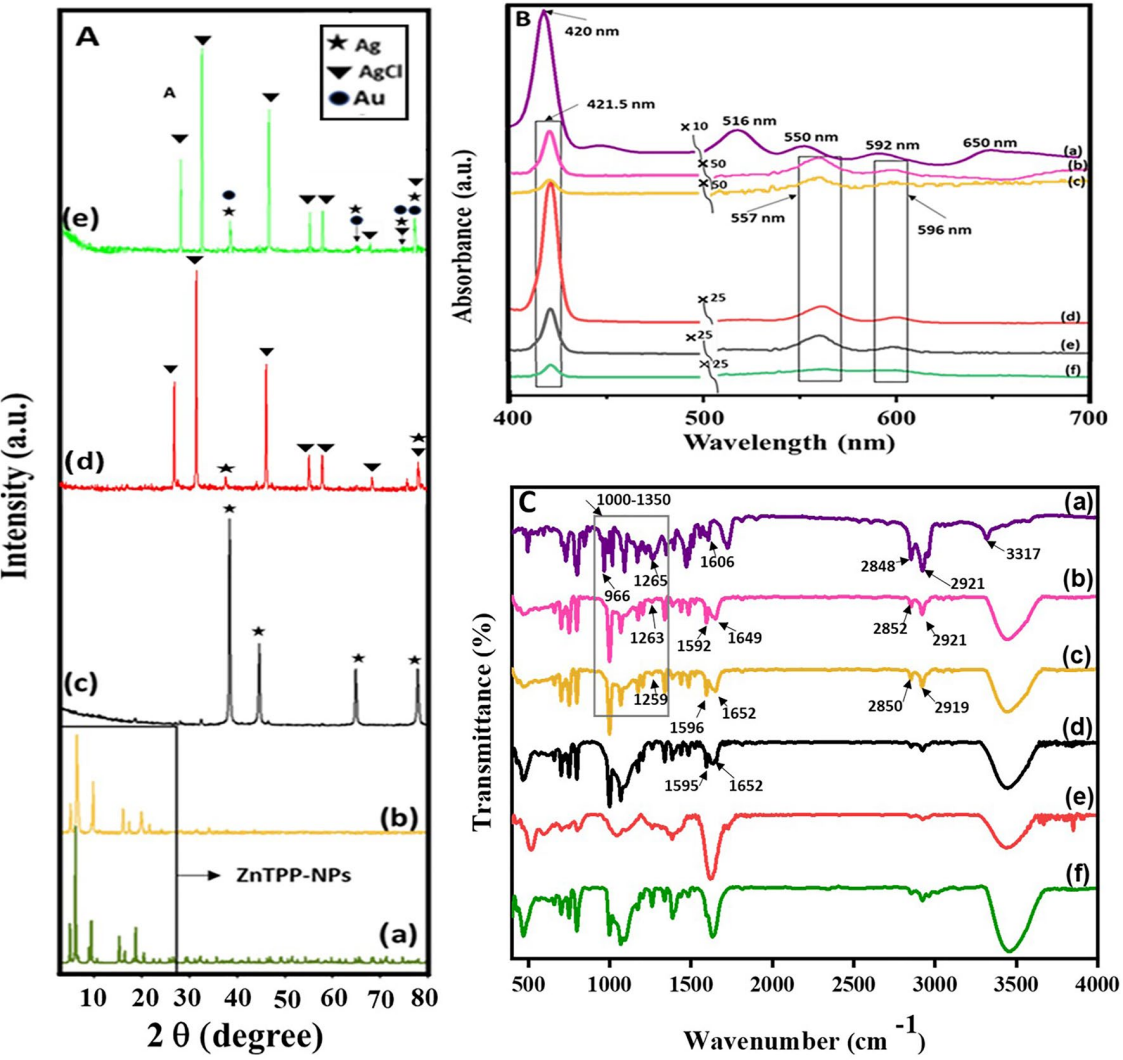
(**A**) PXRD of a simulated pattern of ZnTPP NPs (a), ZnTPP NPs (b)^42^, ZnTPP/Ag NCs (c), ZnTPP/Ag/AgCl/Cu NCs (d), ZnTPP/Au/Ag/AgCl NCs (e), (**B**): UV–Vis spectra of H_2_TPP (a), ZnTPP (b), ZnTPP NPs (c)^42^, ZnTPP/Ag/AgCl NCs (d), ZnTPP/Ag NCs (e), ZnTPP/Au/Ag/AgCl NCs (f), (**C**): FT-IR spectra of H_2_TPP (a), ZnTPP (b), ZnTPP NPs (c)^42^, ZnTPP/Ag NCs (d), ZnTPP/Ag/AgCl/Cu NCs (e), ZnTPP/Au/Ag/AgCl NCs (f).

As shown in Fig. 2Ab the powder X-ray diffraction pattern of self-assembled ZnTPP (ZnTPP NPs) revealed the diffraction peaks at (001), (110), (200), (112), (020), (201), (400), (130) corresponding to the data from the published crystal structure (CCDC, Ref. code ZNTPOR03) octahedrons simulated which was shown in Fig. 2Aa ^28^. In the XRD pattern of ZnTPP/Ag NCs (Fig. 2Ac) four sharp peaks were indicated at 2θ = 38.24°, 44.44°, 64.69°, 77.64° corresponding to (111), (200), (220), and (311) crystalline surfaces of Ag nanoparticles, respectively^29,30^. Figure 2Ad demonstrates the XRD pattern which was attributed to the ZnTPP/Ag/AgCl/Cu NCs. The peaks at 27.96°, 32.40°, 46.40°, 54.96°, 57.64° can be assigned to the (111), (200), (220), (311), (222), (400) AgCl crystalline surfaces^31^. Additionally, the peaks at 2θ = 38.32°, 44.40°, and 77.64° were attributed to the (111), (220), (111) planes of metallic Ag nanoparticles^32^. Since the amount of copper in the synthesized ZnTPP/Ag/AgCl/Cu NCs was below 5% by weight according to the EDS analysis, the peaks of copper are not seen in the XRD pattern of the sample (Fig. 2Ad).

In the XRD pattern that was shown in Fig. 2Ae, the peaks at 29.68°, 56.68°, and 59.32° specified (111), (311), and (222) AgCl reflection planes. Moreover, the peaks at 39.96°, 48.12°, and 78.66° can be attributed to (111), (220), and (311) crystalline surfaces of Au and Ag nanoparticles^36^. Thus considered the formation of ZnTPP/Au/Ag/AgCl NCs. Toward the X-ray diffraction pattern of ZnTPP/Ag NCs, ZnTPP/Ag/AgCl/Cu NCs, and ZnTPP/Au/Ag/AgCl NCs, likely after the formation of nanocomposites, the alternation of the crystalline surfaces and morphology of ZnTPP NPs was seen. The electronic absorption spectroscopic study for the metal-free porphyrin (H_2_TPP) has revealed the typical Soret and Q bands at 420, 516, 550, 592, and 560 nm respectively (Fig. 2Ba). The Soret band is an intense band ranging around 400 nm which is common for all porphyrins. Moreover, the weaker bands in the region of 500–600 nm that are quadruple bands for porphyrin free base and double bands for metalloporphyrins are Q bands. At zinc coordination and metal complex formation, the Soret band at 421.5 nm and two weak Q bands at 557 and 596 nm appeared. Additionally, after metal complexation, a red shift in the Soret band occurred. The molecular symmetry increasing from D_2h_ to D_4h_ led to a change in the number of Q bands from four to two in the metal complex which confirmed the ZnTPP formation (Fig. 2Bb). The UV–Vis spectrum for aggregated ZnTPP (ZnTPP NPs) is demonstrated in Fig. 2Bc. The broad bands significantly are observed. It seems the existence of metal–ligand coordination and π-π stacking interactions involve the tuning of aggregations. Furthermore, in the electronic absorption spectra of other aggregated nanostructure compounds, ZnTPP/Ag/AgCl/Cu NCs, ZnTPP/Ag NCs, and ZnTPP/Au/Ag/AgCl NCs, the Soret band at 421.5 nm and two weak Q bands at 557 and 596 nm are observed (Fig. 2Bd–f) respectively. As shown in Fig. 2Ca–f, the IR spectra of H_2_TPP, ZnTPP, ZnTPP NPs, ZnTPP/Ag NCs, ZnTPP/Ag/AgCl/Cu NCs, ZnTPP/Au/Ag/AgCl NCs, and were demonstrated respectively. In addition, it is known that the Ag and Au nanoparticles have the absorption peaks in the Soret (around 420 nm) and Q peaks (500–650 nm) region of the metalloporphyrin compounds respectively^37–39^. Therefore, the possibility of the overlap of silver nanoparticles absorption with the Soret peak seems logical in the ZnTPP/Ag NCs, ZnTPP/Ag/AgCl/Cu NCs, ZnTPP/Au/Ag/AgCl NCs.

The observed signals at 2850 and 2921 cm^−1^ in the Infrared spectra of H_2_TPP, ZnTPP, and ZnTPP NPs, and all porphyrinic compounds, assigned to the vibrational stretching mode of aliphatic C–H, and signals at 1265 and 1000–1350 cm^−1^ indicated the vibrational mode of C–N. The presence of the signals at 3317 and 966 cm^−1^ are allotted to the stretching and bending vibration of N–H within the pyrrole ring respectively. Moreover, the nonappearance of these two signals in the spectra of ZnTPP indicates the zinc coordination in the complex. The nonappearance of these two signals in the spectra of ZnTPP indicates the zinc coordination in the complex. Moreover, regarding the spectrum of H_2_TPP, the signal at 1606 cm^−1^, which is attributed to the C=N stretching vibration of pyrrole, splits into two peaks at around 1595 and 1652 cm^−1^ indicating the presence of the Zn–N coordination band in the spectra of the prepared ZnTPP NPs which have shifted to 1630 and 1750 nanocomposites formation^24,40–42^.

**Figure 3:**
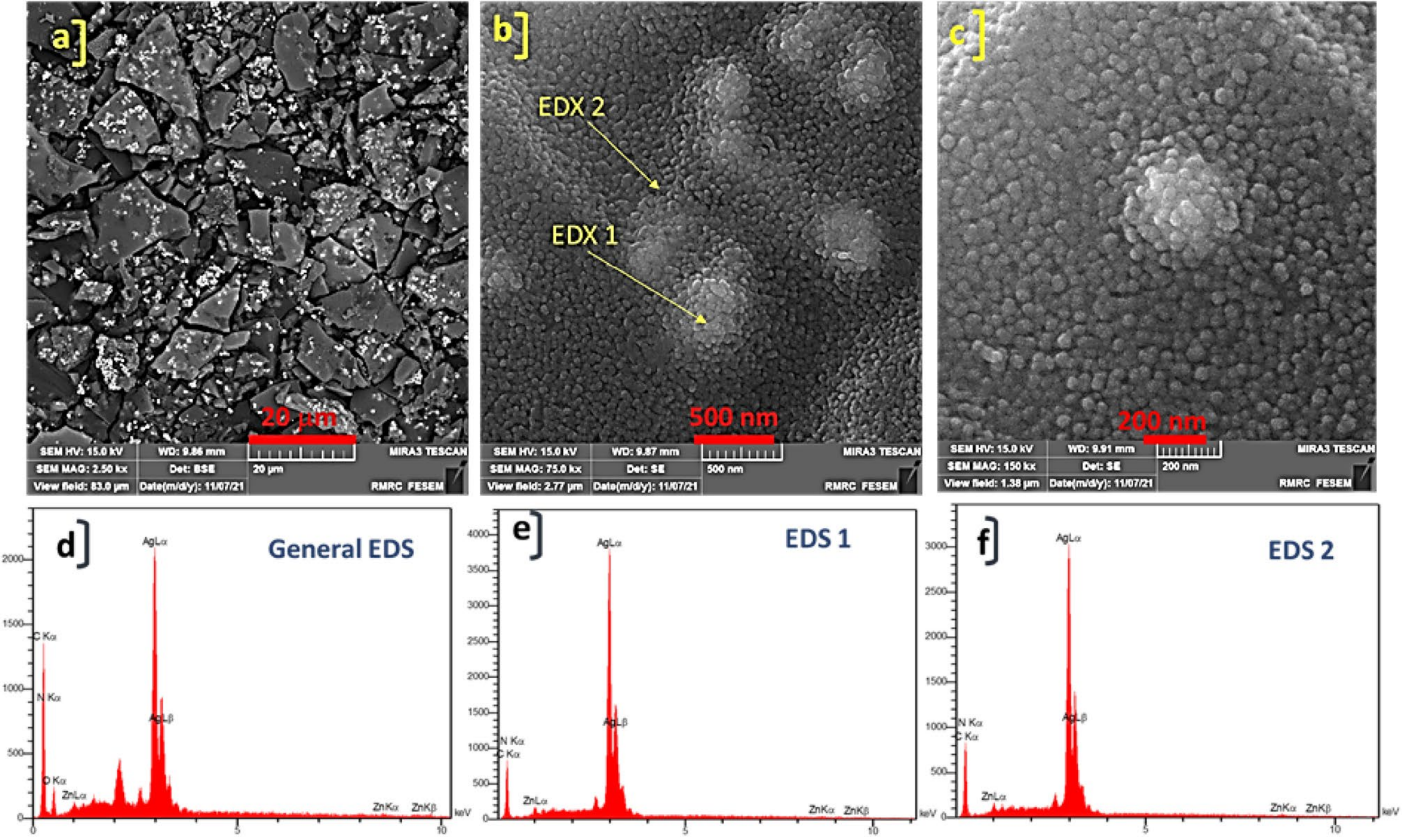
The FE-SEM image of ZnTPP/Ag NCs (**a**–**c**) and the general and pointwise EDS spectra of ZnTPP/Ag NC (**d**–**f**).

**Figure 4:**
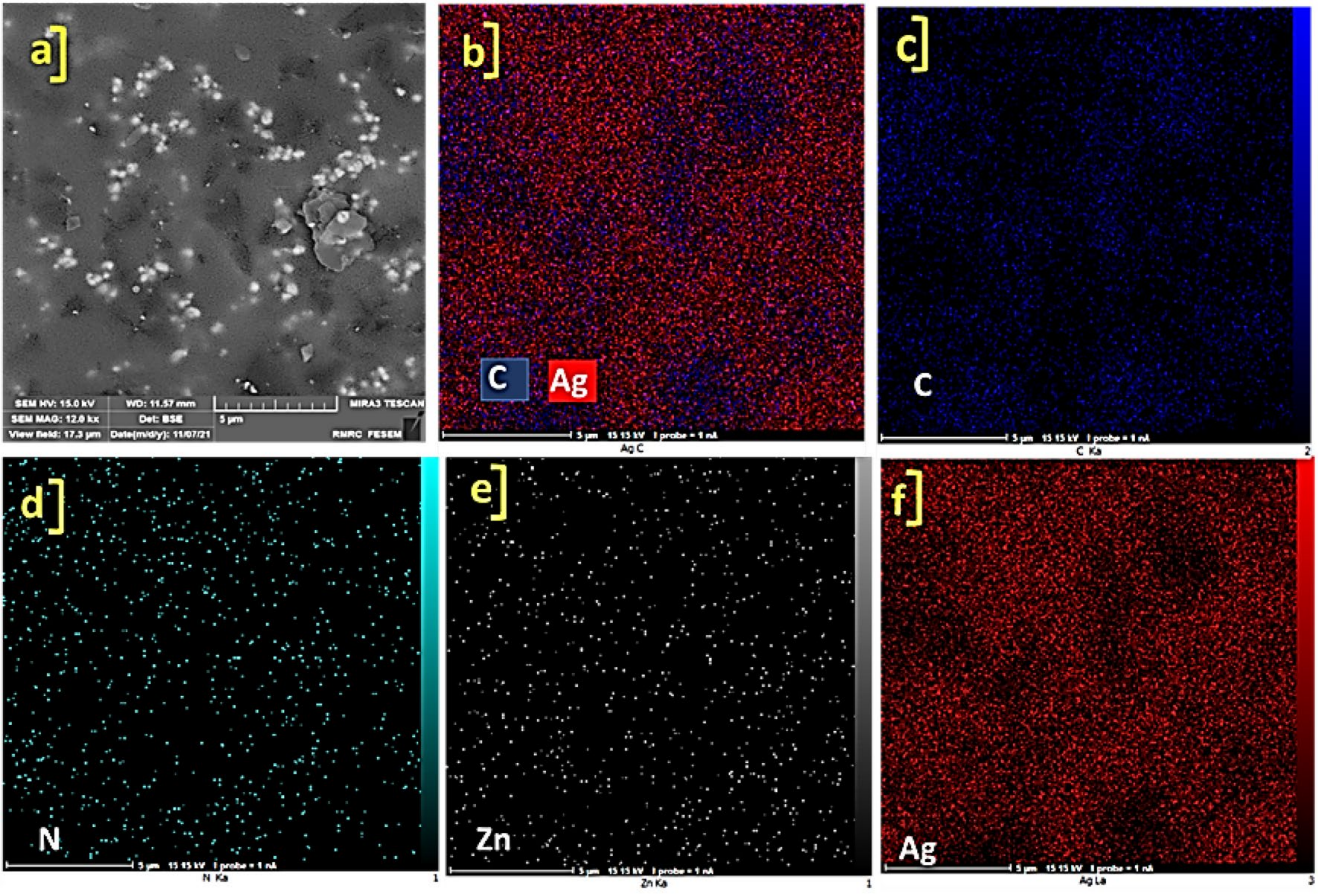
Elemental mapping spectra of ZnTPP/Ag NCs (**a**–**f**).

**Figure 5:**
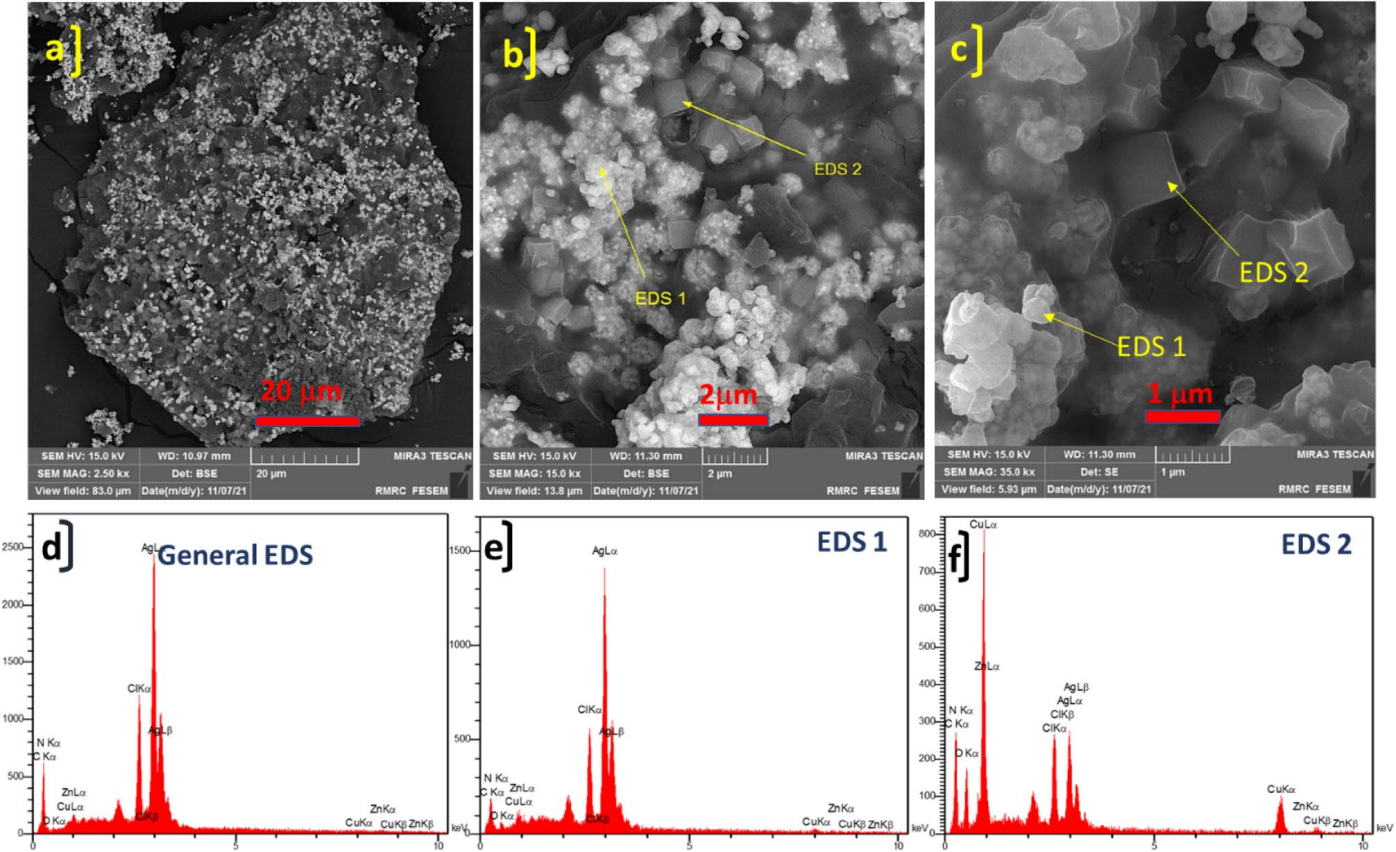
The FE-SEM image of ZnTPP/Ag/AgCl/Cu NCs (**a**–**c**) and the general and pointwise EDS spectra of ZnTPP/Ag/AgCl/Cu NC (**d**–**f**).

**Figure 6:**
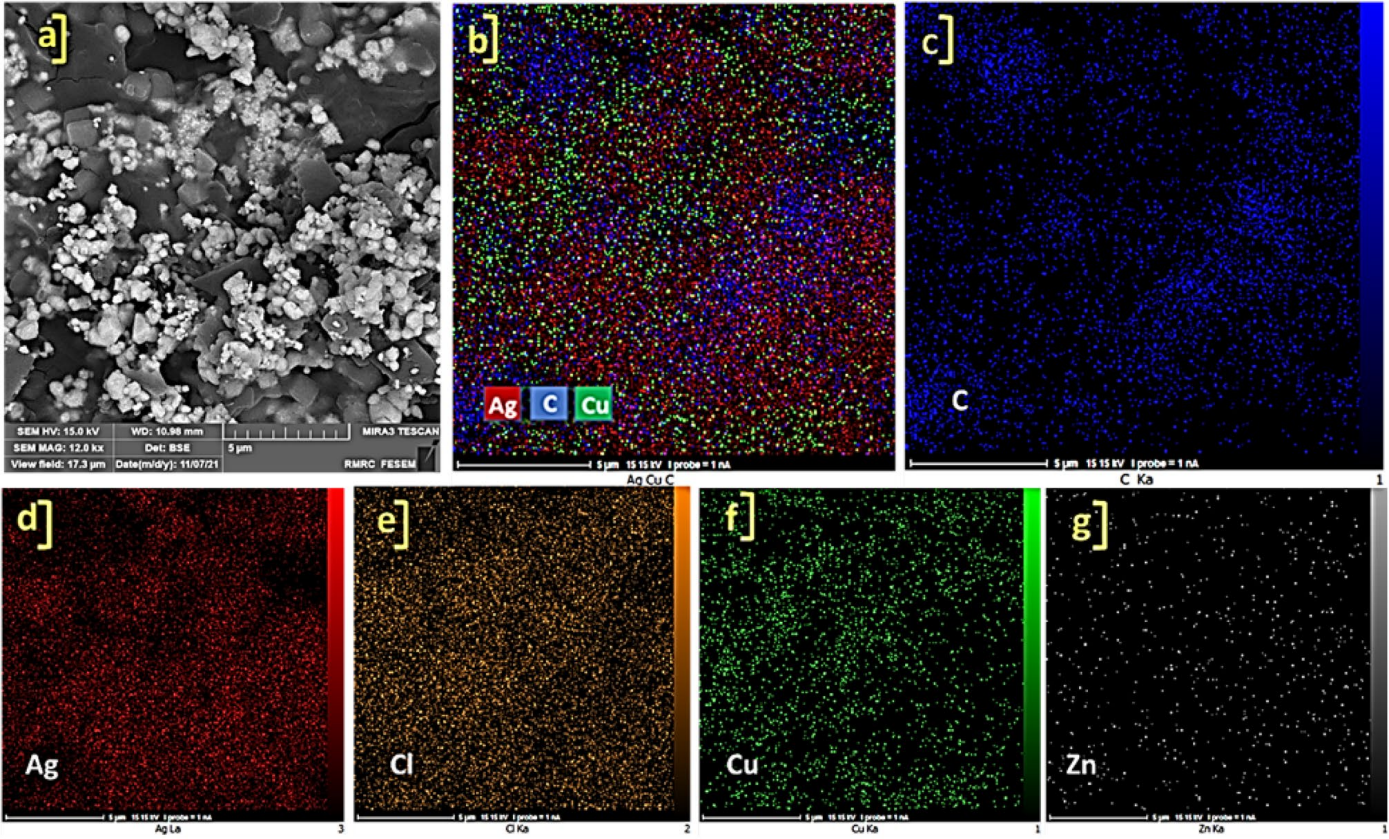
Elemental mapping spectra of ZnTPP/Ag/AgCl/Cu NCs (**a**–**g**).

**Figure 7:**
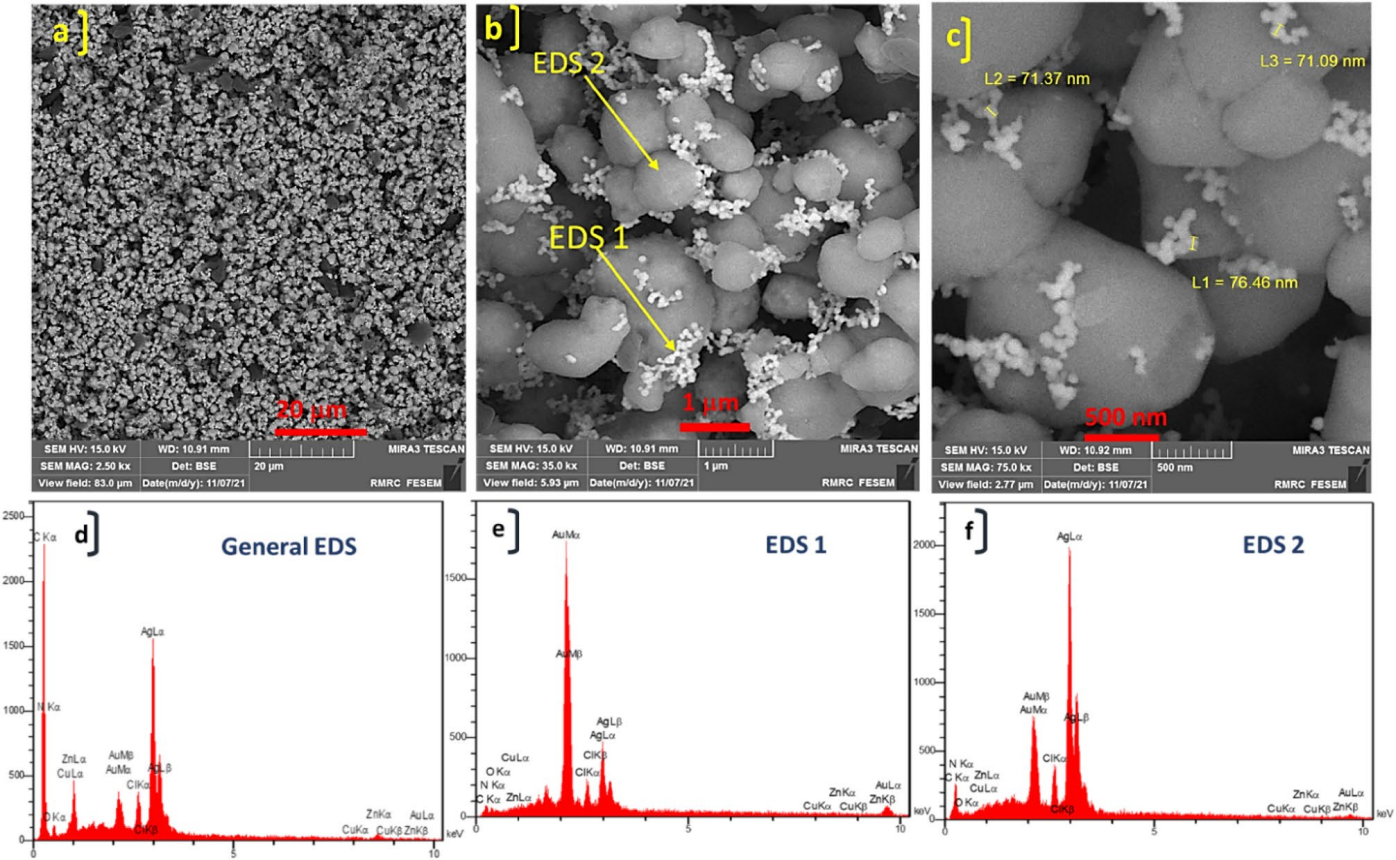
The FE-SEM image of ZnTPP/Au/Ag/AgCl NCs (**a**–**c**) and the general and pointwise EDS spectra of ZnTPP/Au/Ag/AgCl NCs (**d**–**f**).

**Figure 8:**
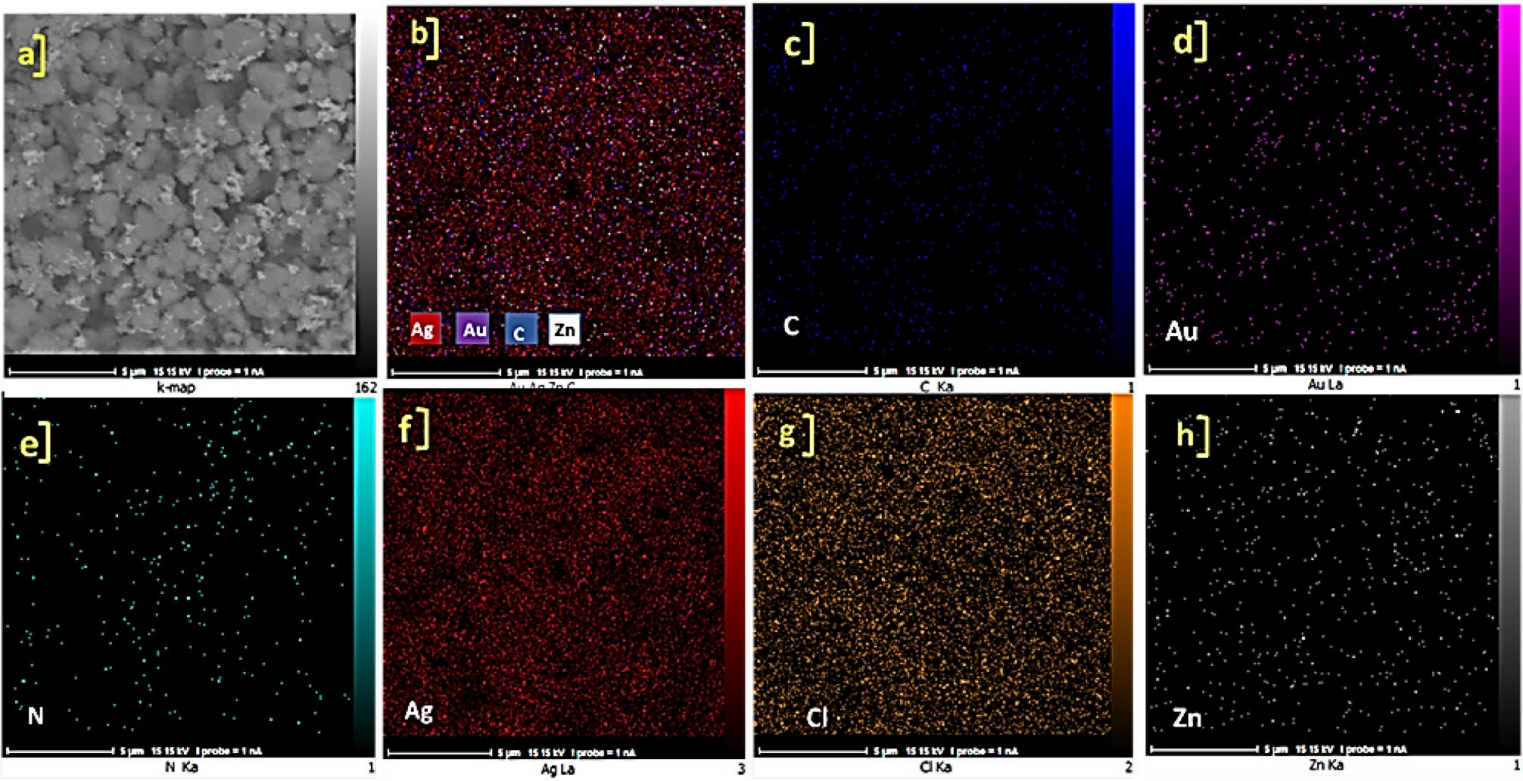
Elemental mapping spectra and of ZnTPP/Au/Ag/AgCl NCs (**a**–**h**).

The FE-SEM images of synthesized ZnTPP NPs were demonstrated in Supplementary Fig. S1 which confirms the octahedral morphology of ZnTPP NPs. Figure 3a–c shows the FE-SEM images of ZnTPP/Ag NCs. The existence of the sphere form of Ag nanoparticles was illustrated. The general and pointwise Energy-dispersive X-ray spectroscopy (EDS) was accomplished (Fig. 3d–f). Moreover, in Fig. 4a–f the elemental mapping analysis of prepared ZnTPP/Ag NCs is shown indicating the presence of Ag NPs with a specified distribution. The FE-SEM images and EDS analysis of synthesized ZnTPP/Ag/AgCl/Cu NCs were shown in Fig. 5a–c and Fig. 5d–f respectively. In the morphological investigation of nanostructures, the cubic-like of particles are seen which is attributed to the Cu compounds in matching with the elemental mapping analysis of prepared ZnTPP/Ag/AgCl/Cu NCs (Fig. 6a–g). Furthermore, other nanoparticles obtained Ag district are attributed to the existence of Ag and AgCl nanoparticles concluded compared with the results of the EDS and XRD analysis. In the FE-SEM images of ZnTPP/Au/Ag/AgCl NCs, the presence of Au nanoparticles is seen (Fig. 7a–c). The obtained general and pointwise EDS analysis confirm the particular districts which contain AgCl and Au nanoparticles individually (Fig. 7d–f). The specific distribution of nanoparticles has been specified in the elemental mapping analysis of prepared ZnTPP/Au/Ag/AgCl NCs (Fig. 8a–h).

**Figure 9:**
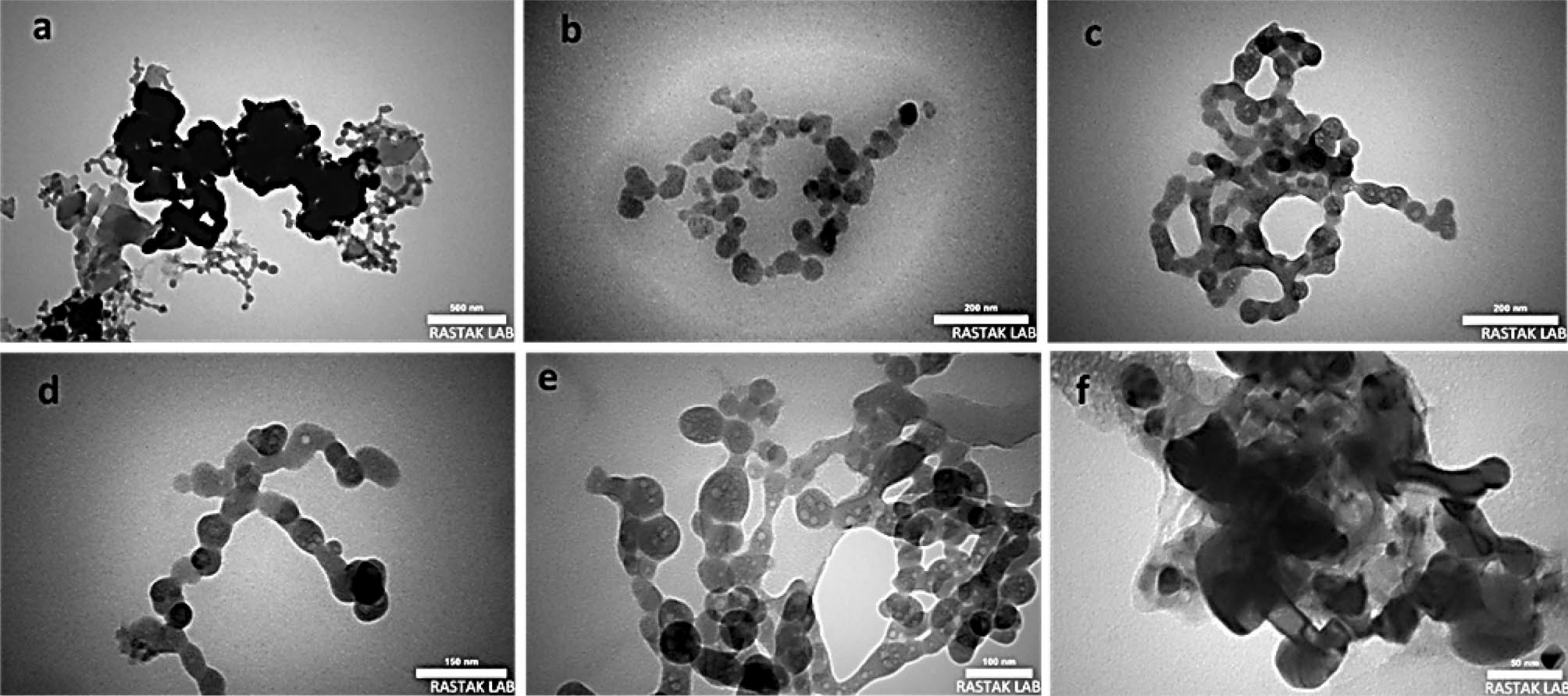
TEM images of ZnTPP/Ag/AgCl/Cu NCs in the scale of 500 nm (**a**), 200 nm (**b**,**c**), 150 nm (**d**), 100 nm (**e**), and 50 nm (**f**).

The collection of transmission electron microscopy (TEM) images of ZnTPP/Ag/AgCl/Cu NCs, the synthesized nanocomposite with the best antibacterial performance, led to the investigation of the size and morphology of the particles of this nanocomposite. The nanoparticles with about 5–10 nm are observed interconnected with the nanocomposite structure (Fig. 9a–f). Evaluating the results of the analysis with the obtained FE-SEM and TEM images together confirms the formation of the obtained multi-component nanocomposites.

## Antibacterial activity appraisement of synthesized porphyrin-based nanocomposites

### Procedure for antibacterial studies with nanocomposite

Samples and glassware were sterilized in an autoclave at 121 °C for 15 min before each microbiological test. The average pH was kept at roughly 7.4, which is the physiological pH. The bactericide experiment included Gram-negative and Gram-positive microorganisms such as *S. aureus* (ATCC 25923) and *E. coli* (ATCC 25922). For colony counting tests, MBC and the inhibition zone method, Mueller–Hinton agar-containing plates were employed as a microorganism growth medium, also, Nutrient Broth media was used for MIC, as well as, the 0.5McFarland turbidity as a standard to the antibacterial test. Intracellular reactive oxygen species (ROS) have been proposed to play critical roles in the antimicrobial mechanism. In this regard, the effect of treatments of synthesized ZnTPP/Ag/AgCl NCs on intracellular ROS for *S. aureus* (ATCC 25923) and *E. coli* (ATCC 25922) was investigated by flow cytometry under light and dark as well.

### Antibacterial activity screening (ZOI study)

**Figure 10:**
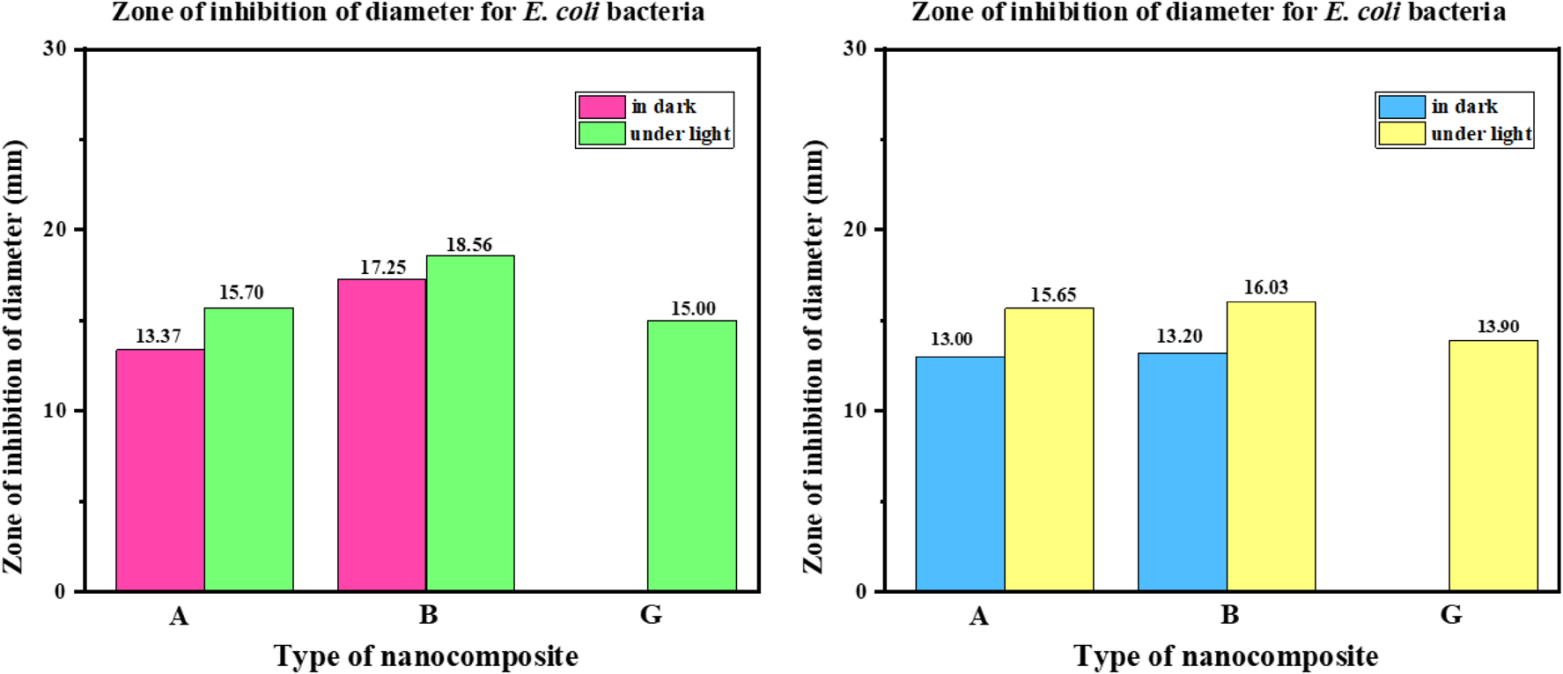
Column charts representing the amounts of the zone of inhibition of diameter for *S. aureus* and *E. coli*, in the presence of A (ZnTPP/Ag NCs), B (ZnTPP/Ag/AgCl/Cu NCs), and G (ZnTPP/Au/Ag/AgCl NCs), in dark and LED light after 24 h.

To investigate the antibacterial activity, the most practicable Agar well diffusion method approach was used. Therefore, the agar well diffusion can be used to test microbial susceptibility to these concentrations of 1 mL (10 mg) of nanocomposites in the presence of these two pathogen bacteria at 37 °C for 24 h, Muller-Hinton agar was used as a foundation medium as well as a solid growth media for microorganisms. First, 0.5 McFarland turbidity suspensions of these two bacteria were generated. About 20 ccs of the medium are put aseptically into sanitized Petri dishes after cooling.using a sterile glass hockey stick, the culture (cell concentration was adjusted to 10^7^ cells/mL) was dispersed across the surface of the solidified agar plates. Correspondingly (Supplementary Fig. S2). The ZOI (zones of inhibition) surrounding the circle-tested samples assess their antibacterial efficacy against two important pathogens, *E. coli*, and *S. aureus*. The obtained zones of inhibition of diameter are represented in Supplementary Table S1. The results of the zones of inhibition of diameter for *E. coli* and *S. aureus* in dark and under light conditions were represented in column charts in Fig. 10.

### MIC and MBC determination

**Figure 11:**
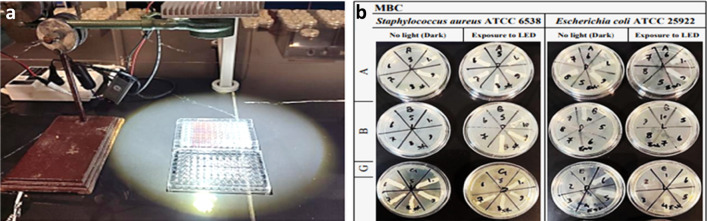
(**a**) Photographs of the 96-well MIC microplates and (**b**) MBC plates for *E. coli* and *S. aureus* in the presence of nanocomposites exposed to LED.

In this study, For the first time, we selected two 96-well microplates for the study of MIC and MBC with LED light and dark on two types of pathogenic bacteria E.coli and S. aureus, in the presence of ZnTPP/Ag NCs (A), ZnTPP/Ag/AgCl/Cu NCs (B), and ZnTPP/Au/Ag/AgCl NCs (G), with serial dilution method, considering that the first well had the highest concentration of 5000 ppm (µg/mL) and Finally, we sorted the last well with the lowest concentration of 9.7 ppm (µg/mL) nanocomposite and finally added (1 × 10^5^ CFU/mL)bacteria to each well to the Muller Hinton Broth (MHB) culture medium in both 96-well microplates. One well always had only the culture medium containing bacteria to control. Then one 96-well microplate was placed in LED light for 2 h and another microplate was placed in the dark for 2 h, then the microplate was incubated for about 18 h at 37 °C, then from 6 houses that had a clear Broth culture in order Determination of concentration of nanocomposites or other words MIC and MBC were poured on a plate containing Casein-peptone Soymeal-peptone Agar (CASO Agar) and after 24 h at 37 °C incubator (Fig. 11a,b, Supplementary Figs. S3, S4) respectively.

**Figure 12:**
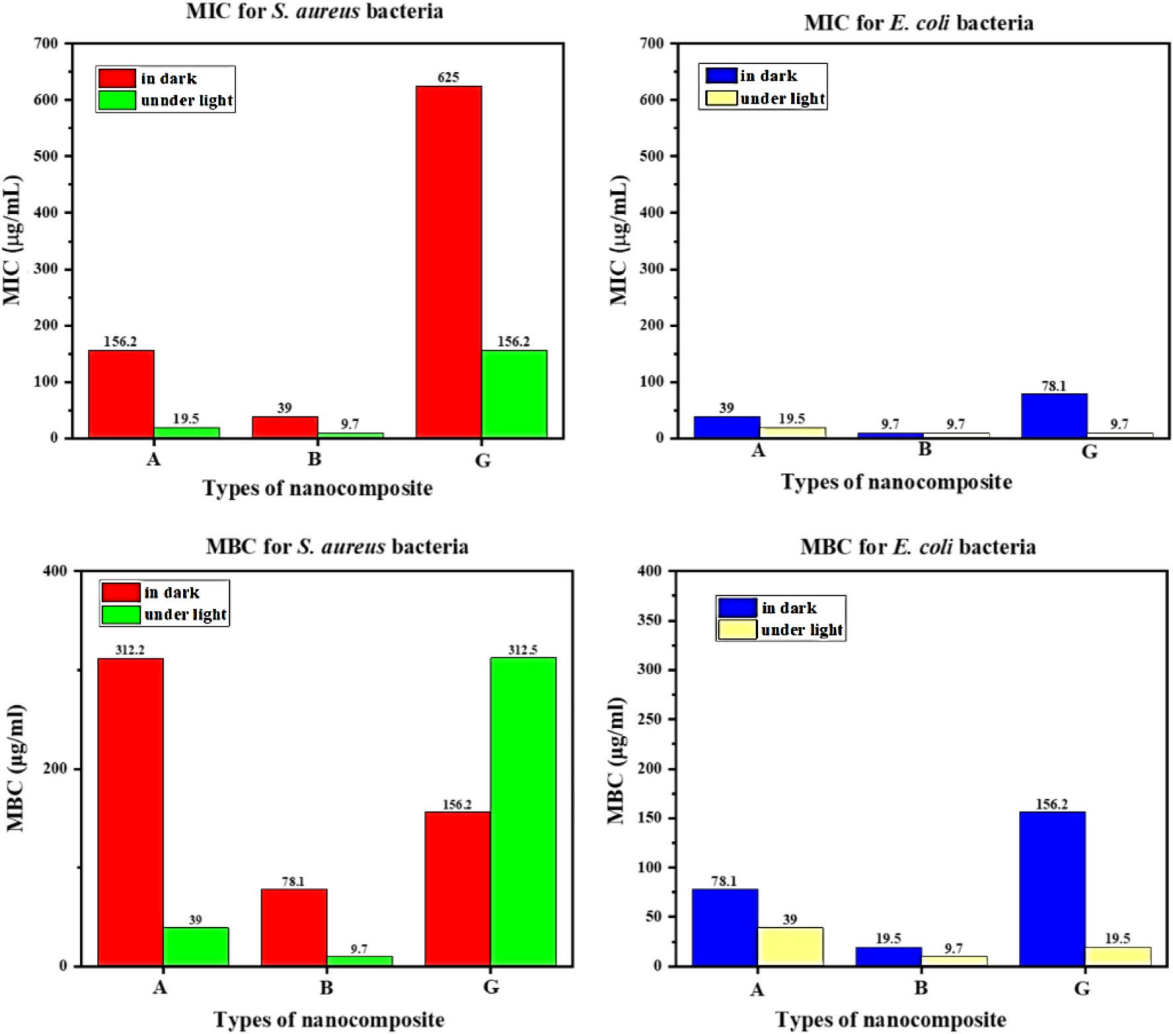
Column charts representing the MIC and MBC values of nanocomposites (A, B, and G) for *S. aureus* (ATCC 25923) and *E. coli* (ATCC 25922) in dark and after 2 h exposure to LED light.

The MIC test results in dark and under light conditions for *E. coli* and *S. aureus* were evaluated in Supplementary Tables S2, and S3 respectively. A well microplate in which bacteria did not grow as MBC means and the lowest bactericidal concentration of nanocomposite bacteria were selected and the microplate house before MBC was selected as the highest bactericidal inhibitory concentration MIC. Figure 11b, Supplementary Fig. S4, and Supplementary Tables S4, and S5 represent the MBC plates and their result values respectively. Finally, ZnTPP/Ag/AgCl/Cu NCs (B) nanocomposite had the highest antibacterial potency compared to ZnTPP/Ag NCs (A) and ZnTPP/Au/Ag/AgCl NCs (G) nanocomposites. Both pathogenic bacteria were reported as gram-positive and gram-negative. Samples A and B were observed in the LED light exposure with more antibacterial properties than in the dark state and G nanocomposites in the dark state with more antibacterial power than the LED light irritation. Figure 12 demonstrates the results of the obtained MIC and MBC values for *S. aureus* (ATCC 25923) and *E. coli* (ATCC 25922) in dark and after 2 h exposure to LED light.

### Plate-count method

**Figure 13:**
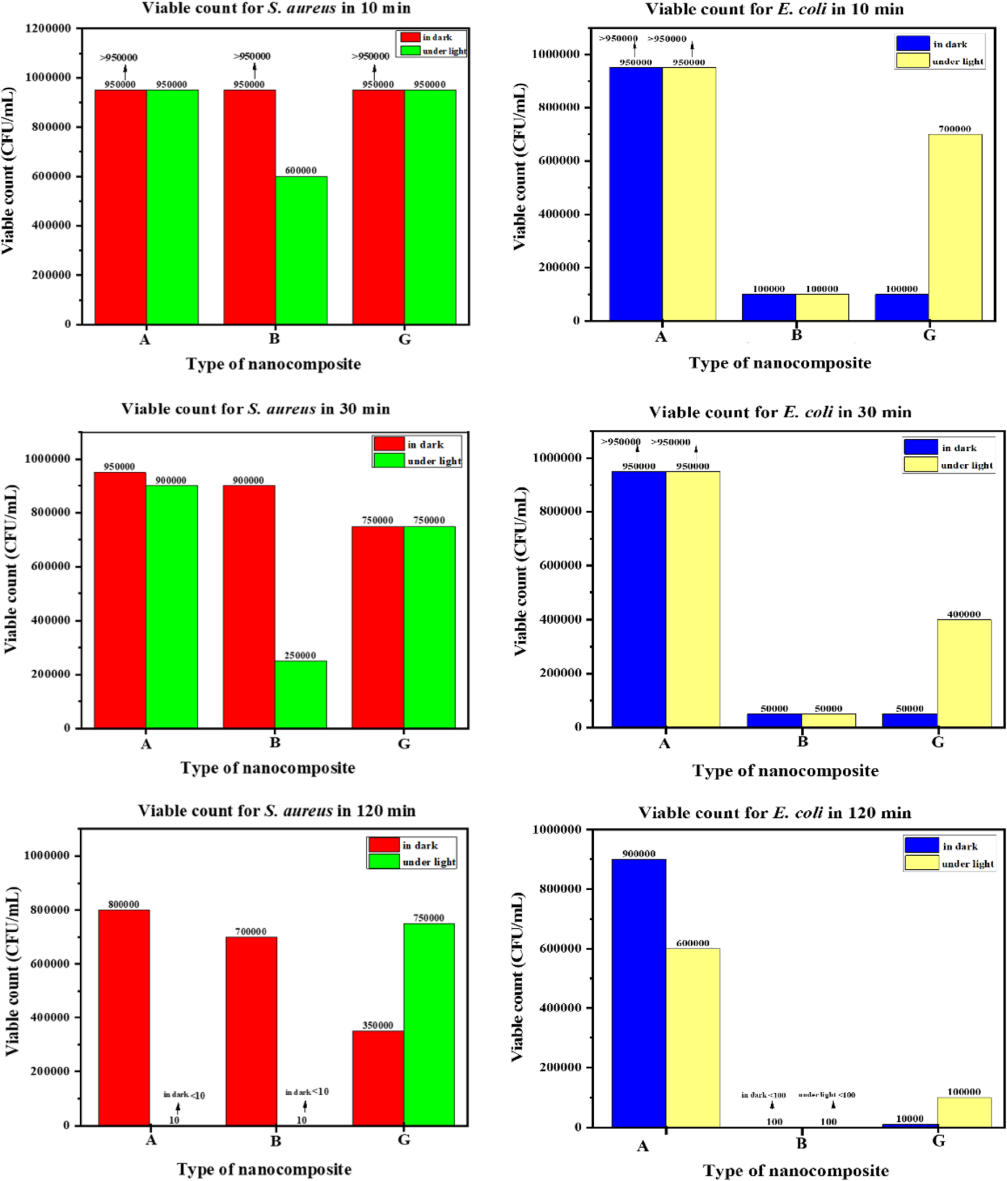
Column charts representing the results of the viable counts tests for *S. aureus* (ATCC 25923) and *E. coli* (ATCC 25922) in dark and under LED light after 10, 30, and 120 min.

The adjustment was made to a diluted 0.5 McFarland turbidity standard of *Staphylococcus aureus* and *Escherichia coli*. For the colony count procedure, 0.1 mL of DMSO was added to Mueller Hinton Broth culture media. The acquired opacity solution was then divided into two portions for each bacterium and placed in two different flasks. Nanocomposite (0.01 g) was added to one of the solutions under LED illumination and the remaining flask has in dark. Then the obtained results for *S. aureus* and *E. coli* in dark and under light conditions were fully evaluated in Supplementary Tables S6, S7, S8, and S9 respectively. The Photographs of CFU of *E. coli* and *S. aureus* in the presence and absence of nanocomposites with LED light and dark after 10, 30, and 120 min are shown in Supplementary Figs. S5, and S6 respectively^**.**^ According to the results obtained from the viable count tests in the presence of A, B, and G nanocomposites and the very good performance of nanocomposites A and B in exposed to LED lamps than dark mode on the bacterium *E. coli* and *S. aureus*, at times 10 min*,* 30 min and 2 h can be mentioned according to the column charts which have been represented in Fig. 13.

**Figure 14:**
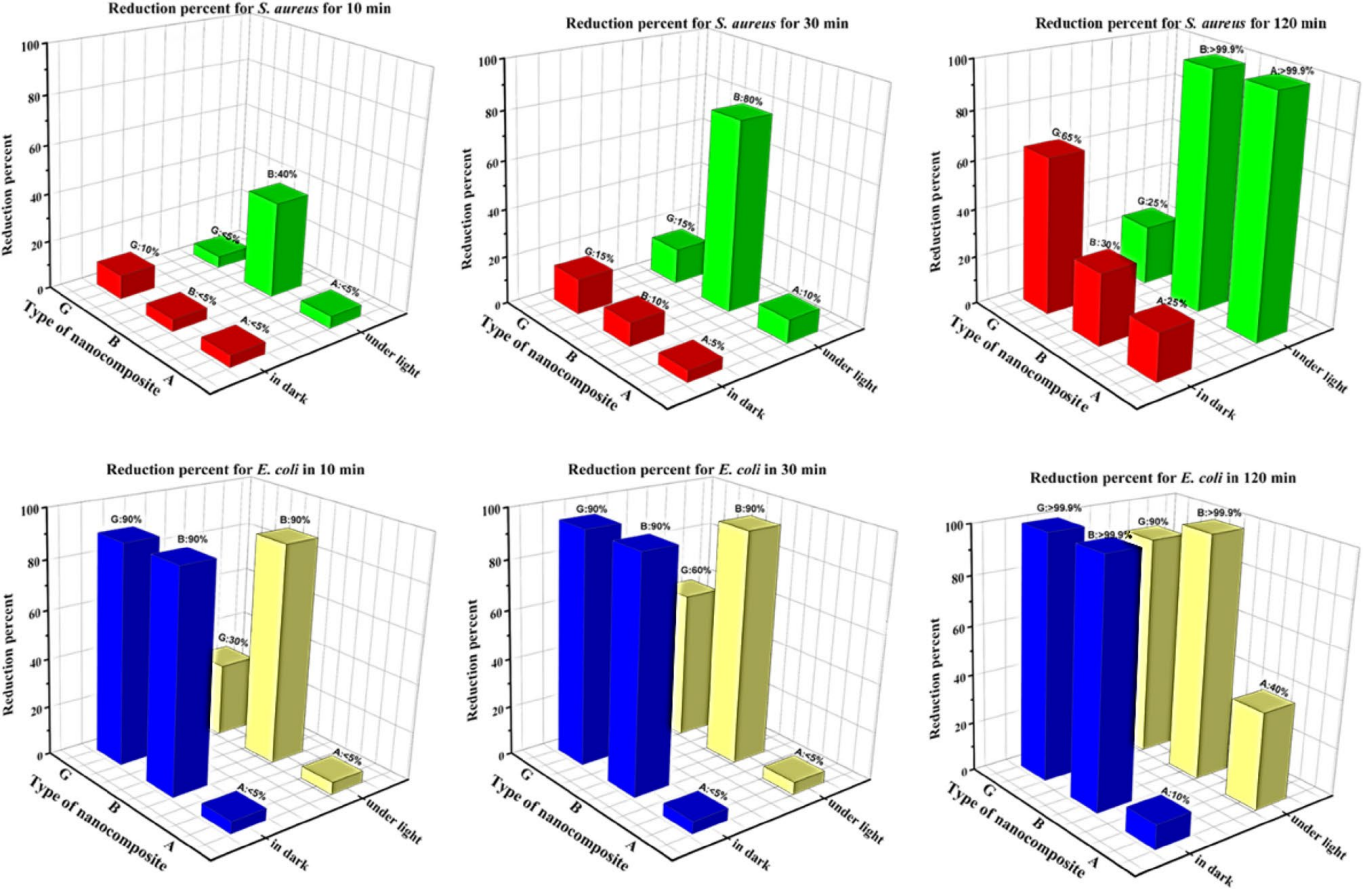
Column charts representing the reduction percentage for *S. aureus* and *E. coli* in dark and under LED light in 10,30, and 120 min.

G nanocomposite was reported to have slightly better antibacterial in the dark than in the light while the performance of B nanocomposite was phenomenal. The colony method also revealed the existence of inactivated (killed) *E. coli* (ATCC 25922) bacteria and *S. aureus* (ATCC 25923) in the presence of these nanocomposites. Finally, they were found to be effective against two harmful bacteria strains. The reduction percentages for *S. aureus* and *E. coli* were expressive of the antibacterial activity of each nanocomposite in dark and under LED light (Fig. 14).

## ROS measurement by flow cytometry

The detection of the intracellular ROS was done by the general oxidative stress indicator CM-H2DCFDA. DCFDA (10 mL; Sigma, Life Technologies C6827) was aggravated to the cells and incubated at 37 °C for 25 min. The DCFDA fluorescent probe reacts with intracellular H_2_O_2_ to procreate fluorescence emission which can be indicated by flow cytometry. Mensuration of the intracellular H_2_O_2_ production in SACS, ere and since culture, was done by flow cytometry using DCFDA. The cells were washed twice with PBS and then centrifuged at 2500*g* for 5 min. green fluorescence emission was measured between 500 and 530 nm using a flow cytometry machine.

**Figure 15:**
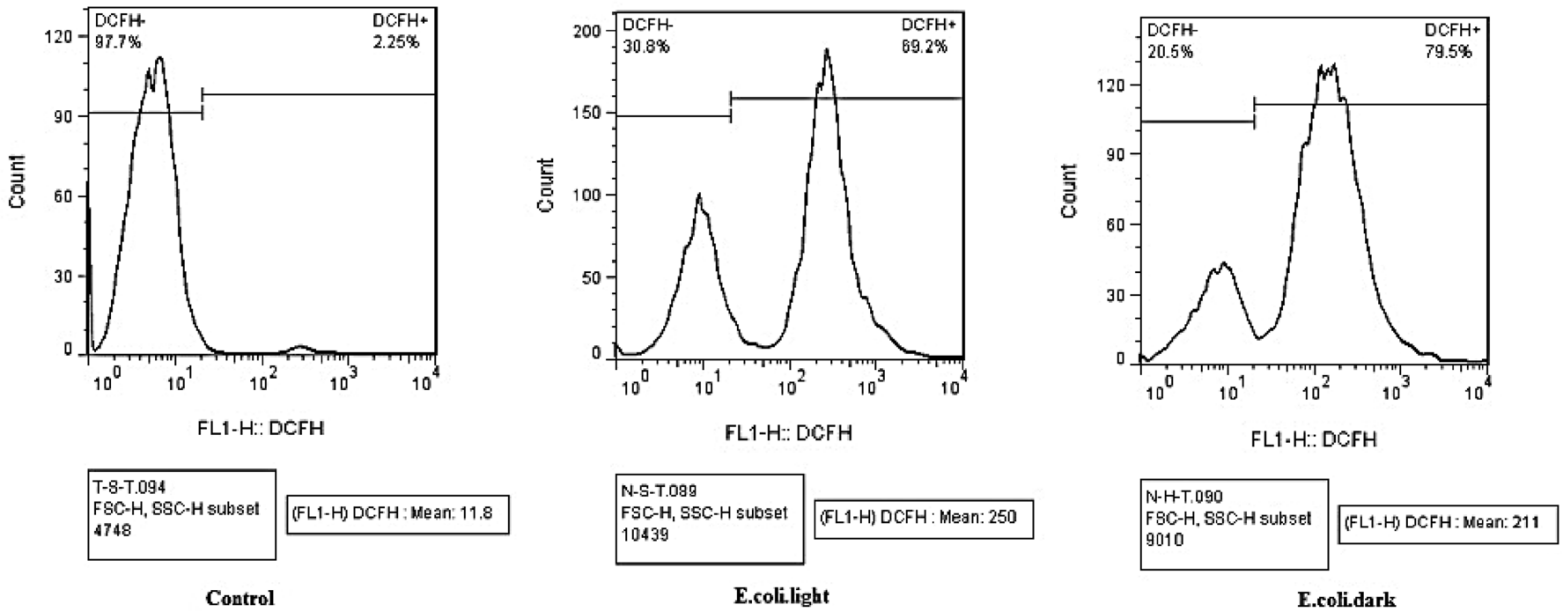
Histograms for ROS production of *E. coli* demonstrating increased fluorescence after treatment with ZnTPP/Ag/AgCl/Cu NCs under light and dark.

**Figure 16:**
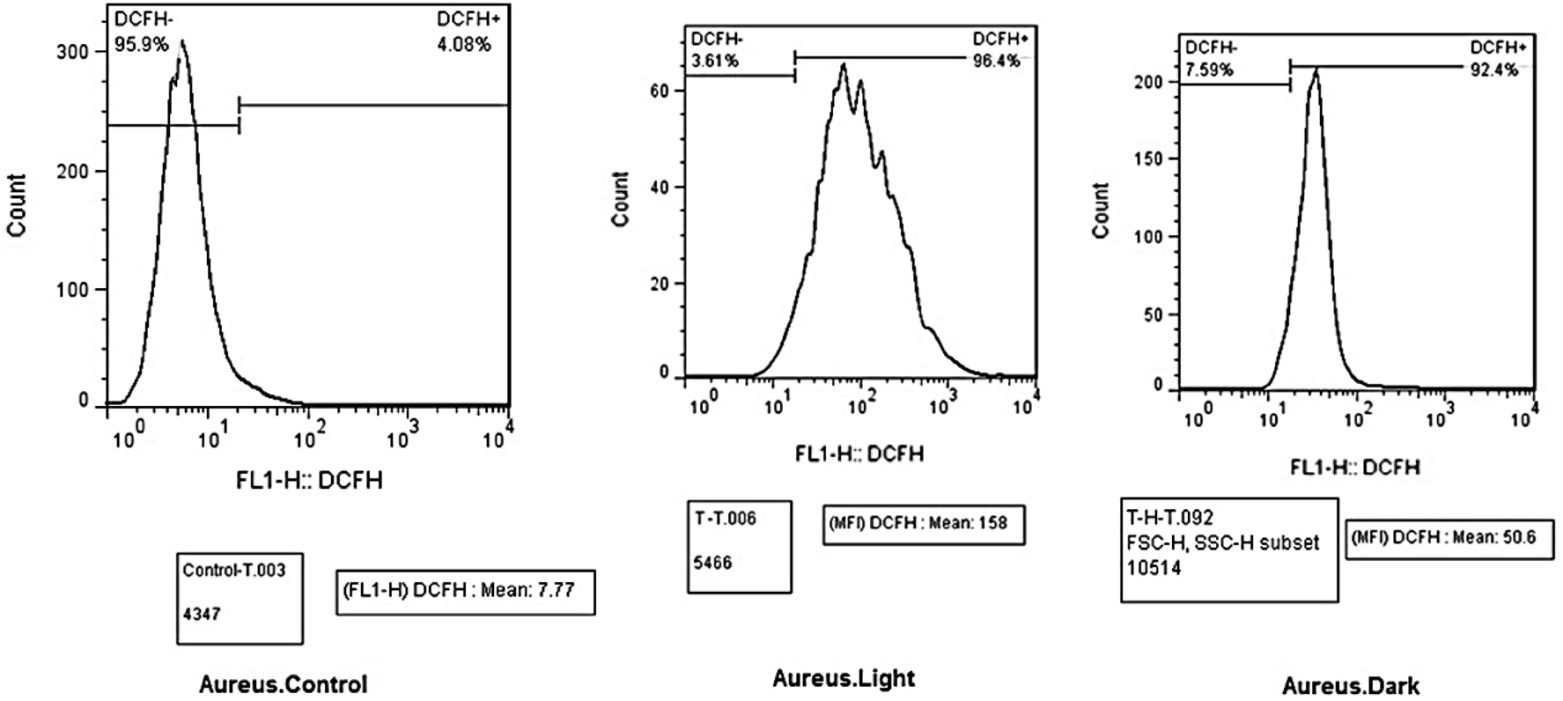
Histograms for ROS production of *S. aureus* demonstrating increased fluorescence after treatment with ZnTPP/Ag/AgCl/Cu NCs under light and dark.

The flow cytometry ROS evaluation was also done under dark and LED light as well. The multi roles of ROS in bacterial replication to antibacterial agents have been confirmed by evidence^43^. Here the results of the measurements of produced intracellular ROS in *E. coli and S. aureus*, after treatment with ZnTPP/Ag/AgCl/Cu NCs have been represented in Figs. 15, and 16 respectively. Considering that the higher amount of fluorescence indicates a more level of ROS production. In the histogram obtained from the flow cytometry analysis, the more the right-side stays, demonstrates the increase of the ROS. The reported MIF value represents the intensity of the color in each sample. For *E. coli* bacteria the ROS increased after treatment with ZnTPP/Ag/AgCl/Cu NCs under both dark and light conditions while more increase under light occurred. For *S. aureus* bacteria, after treatment, the increase in ROS production was obtained in dark and light. Although the under-light enhancement was more than the dark condition for *S. aureus* bacteria as well, however, the surplus increase of ROS for *E. Coli* bacteria was seen under light.

### Mechanism for antibacterial activity of nanocomposite

**Figure 17:**
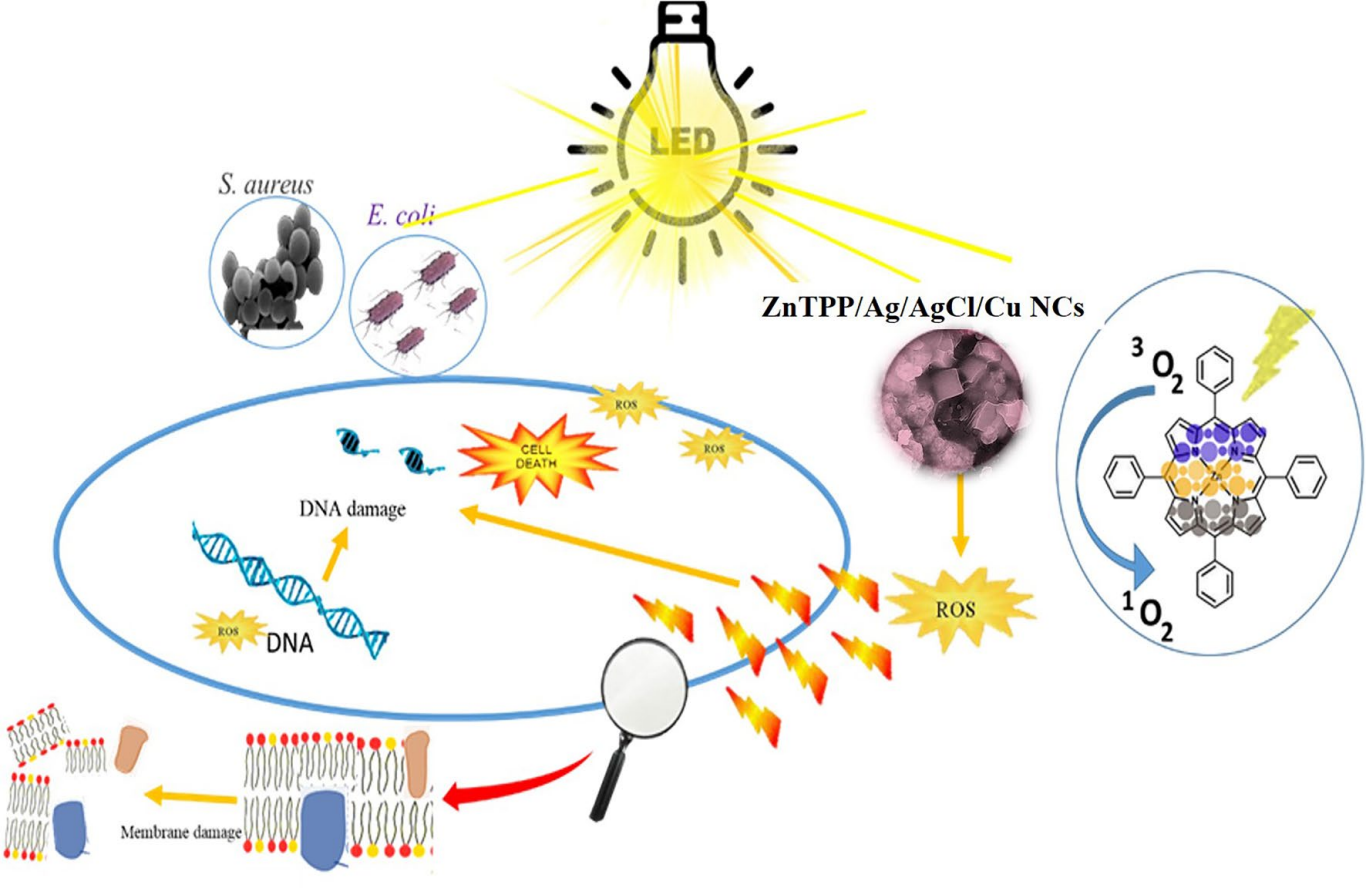
Proposed antibacterial mechanism of the ZnTPP/Ag/AgCl/Cu NCs.

Flow cytometry was used to investigate the mechanism of antibacterial activity of the nanocomposite on *E. coli* and *S. aureus bacteria.* ROS is thought to be a key role in nanomaterial’s antibacterial properties, as it can harm the cell membrane, phospholipids, and/or membrane proteins directly. Among the biologically relevant ROS, superoxide (O_2_^**.−**^) is arising from electron transfer processes^43^. Nanomaterials with the ability to produce reactive oxygen species (ROS) could be a useful tool in the fight against bacteria^44,45^. PDT relies on the production of ^1^O_2_ as the predominant cytotoxic ROS for its lethal effects. It reacts with more than one target within a cell including DNA bases, proteins, and cholesterol found in cell membranes. Porphyrin, as a photosensitizer, could cause the surrounding oxygens to produce reactive oxygen species (ROS) when exposed to a broad spectrum of light^46,47^. Ag and AgCl NPs have antibacterial synergies in ROS production^48,49^. In addition, Because of the visible bang gap of Porphyrin-based nanocomposites, they are favorable compounds to produce superoxide under an LED light. According to the reported article, the synthesized nanocomposite has antibacterial activities on *E. coli* and *S. aureus*, and the attendance of nanocomposite and in the presence of light has been linked to the production of reactive oxygen species (ROS). The bacteria were harmed as a result of ROS generation damaging the membranes (Fig. 17).

## Cell viability assay

**Figure 18:**
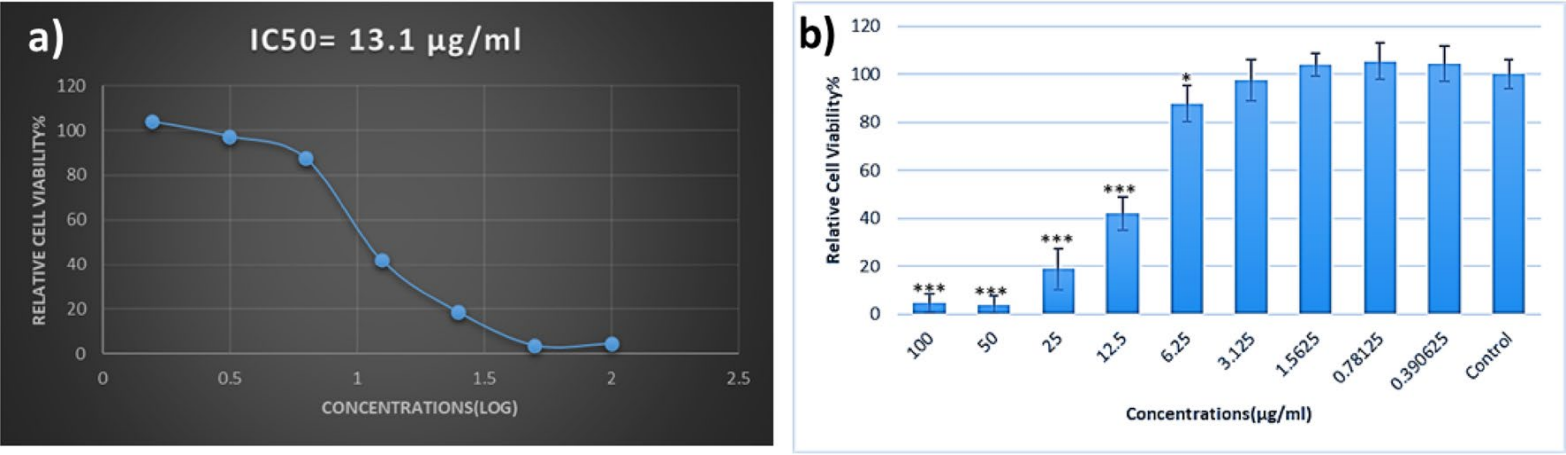
The viability of HFF-1 cells, 24 h after treatment with ZnTPP/Ag/AgCl/Cu NCs.

MTT test was performed to measure the toxicity of ZnTPP/Ag/AgCl/Cu NCs on HFF-1 Human foreskin fibroblast cells. 1 × 104 cells were seeded to each well of a 96‐well plate for 24 h. Different concentrations (0,0.390625, 0.78125, 1.5625, 3.125, 12.5, 25, 50, 100 μg/mL) of ZnTPP/Ag/AgCl/Cu NCs were added to the plate (eight replicates). The plate incubation for 48 h was done. Then, 100 μL of MTT solution (at a final concentration of 0.05 mg/well) was added. Afterward, 100 μL/well of dimethyl sulfoxide (DMSO) was utilized to solubilize the formed formazan crystals in cells. The absorption was read at 570 nm using a microplate reader. The inhibition of HFF-1 cells after 24 h of incubation with the concentrations of ZnTPP/Ag/AgCl/Cu NCs was dependent on doses. The half-maximal inhibitory concentration (IC50) value of ZnTPP/Ag/AgCl/Cu NCs was 13.1 μg/mL for HFF cells in 24 h (Fig. 18). Since the obtained value of IC50 for ZnTPP/Ag/AgCl/Cu NCs compared with the obtained MIC (9.7 μg/mL) is more, it seems this nanocomposite can be utilized in some tracible treatments.

## Conclusions

In summary, novel nanocomposites were synthesized using an effective and green method applied to develop and construct a useful, new, heterogeneous modified ZnTPP with Ag Cu, and Au nanostructures in a short amount of time. The nanocomposite structure, morphology, and antibacterial capabilities have all been evaluated using a variety of analytical methods. Fe-SEM images confirmed the octahedral structure, as well as the great dispersion of, Au, Cu, and Ag NPs on the ZnTPP NPs surface. These nanocomposites also had outstanding antibacterial characteristics in the presence of light. Agar disk diffusions, MBC. MIC, colony counting experiment, and ROS Flow cytometry analysis were used to examine the antibacterial property of these nanocomposites against *E. coli* and *S. aureus*. All of the antibacterial tests and the intracellular ROS evaluation were examined in the dark and under LED light as well. ZnTPP/Ag/AgCl/Cu NCs nanocomposite performed better in the presence of light with a MIC value of less than 9.5 µg/mL, while ZnTPP/Au/Ag/AgCl NCs efficiency of this nanocomposite was better in the dark. The cytotoxicity of ZnTPP/Ag/AgCl/Cu NCs against HFF-1 cells was investigated. According to the findings, these nanocomposites could be used with almost consistent efficiency in water purification and industrial applications. Also, it has the potential to be utilized in biotechnology and therapeutics. The use of such nanocomposites in in vivo curative applications such as photodynamic therapy is thus a promising approach.

## Supplementary Information

Supplementary Information.

## Data Availability

All data generated or analyzed during this study are included in this published article.
