# Supplementary material for: Facile photosynthesis of novel porphyrin-derived nanocomposites containing Ag, Ag/Au, and Ag/Cu for photobactericidal study
Source: Sci Rep. 2023 May 26;13:8580. doi: 10.1038/s41598-023-34745-0 (PMC10220001; doi:10.1038/s41598-023-34745-0)
Supplement: Supplementary file 1 — Supplementary Information. [file 41598_2023_34745_MOESM1_ESM.docx]

**Electronic Supporting Information**

**Facile photosynthesis of novel porphyrin-derived nanocomposites containing Ag, Ag/Au, and Ag/Cu for photobactericidal study**

Sajedeh Tehrani Nejad, ^a^ Rahmatollah Rahimi, *^,a^ Mahboubeh Rabbani, ^a^ Sadegh Rostamnia *^,b^

*^a^ Inorganic Group, Department of Chemistry, Iran University of Science and Technology (IUST), 16846-13114, Tehran, Iran. Email:* [*rahimi_rah@iust.ac.ir*](mailto:Rahimi_rah@iust.ac.ir)

*^b^ Organic and Nano Group (ONG), Department of Chemistry, Iran University of Science and Technology (IUST), PO Box 16846-13114, Tehran, Iran. Email: rostamnia@iust.ac.ir*

**
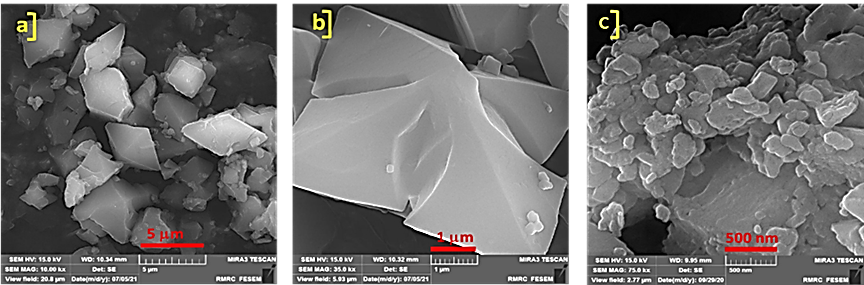
**

**Figure S1.** (a-c) The FE-SEM image of ZnTPP-NPs (for more SEM of ZnTPP-NPs after freeze-drying and before of freeze-drying see ref. 1).^1^

**Table S1.** Average zone of inhibition of samples (mm). (Three reputations)

**
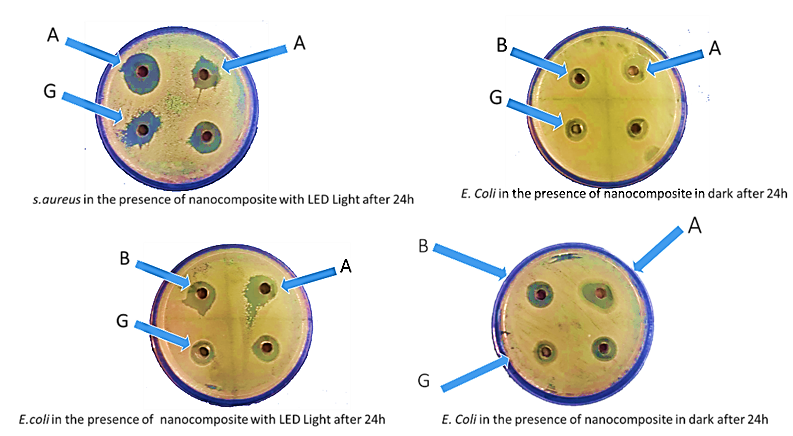
**

**Figure S2.** Images of ZOI of a) *S. aureus*, b) *E. coli*, in the presence of A (ZnTPP/Ag-NPs), B (ZnTPP/Ag/AgCl/Cu-NPs), and G (ZnTPP/Au/Ag/AgCl-NPs), in dark and LED light after 24h.


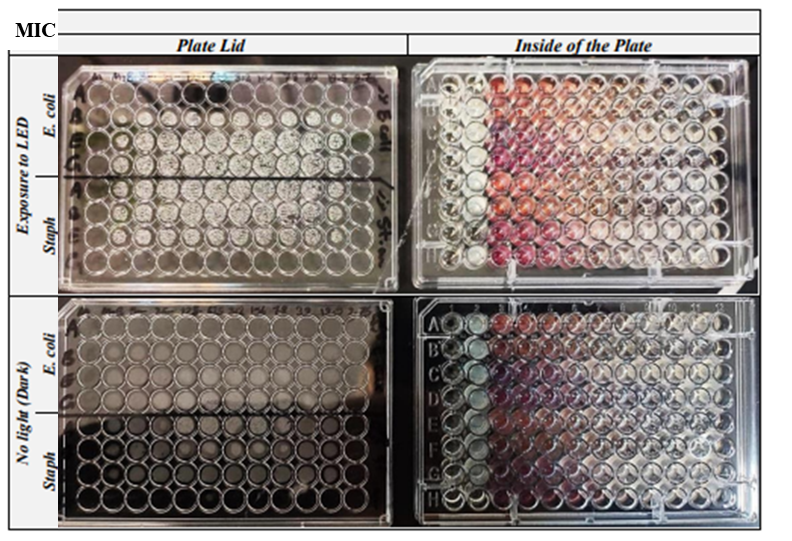


**Figure S3.** Photographs of the 96-well microplates of *E. coli* and *S. aureus* in the presence of nanocomposites.


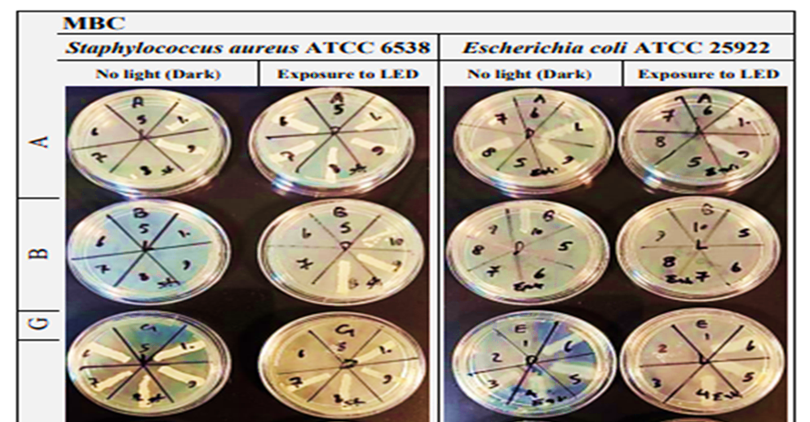


**Figure S4.** The MBC plates for *S. aureus* and *E. coli* in dark and under light conditions.


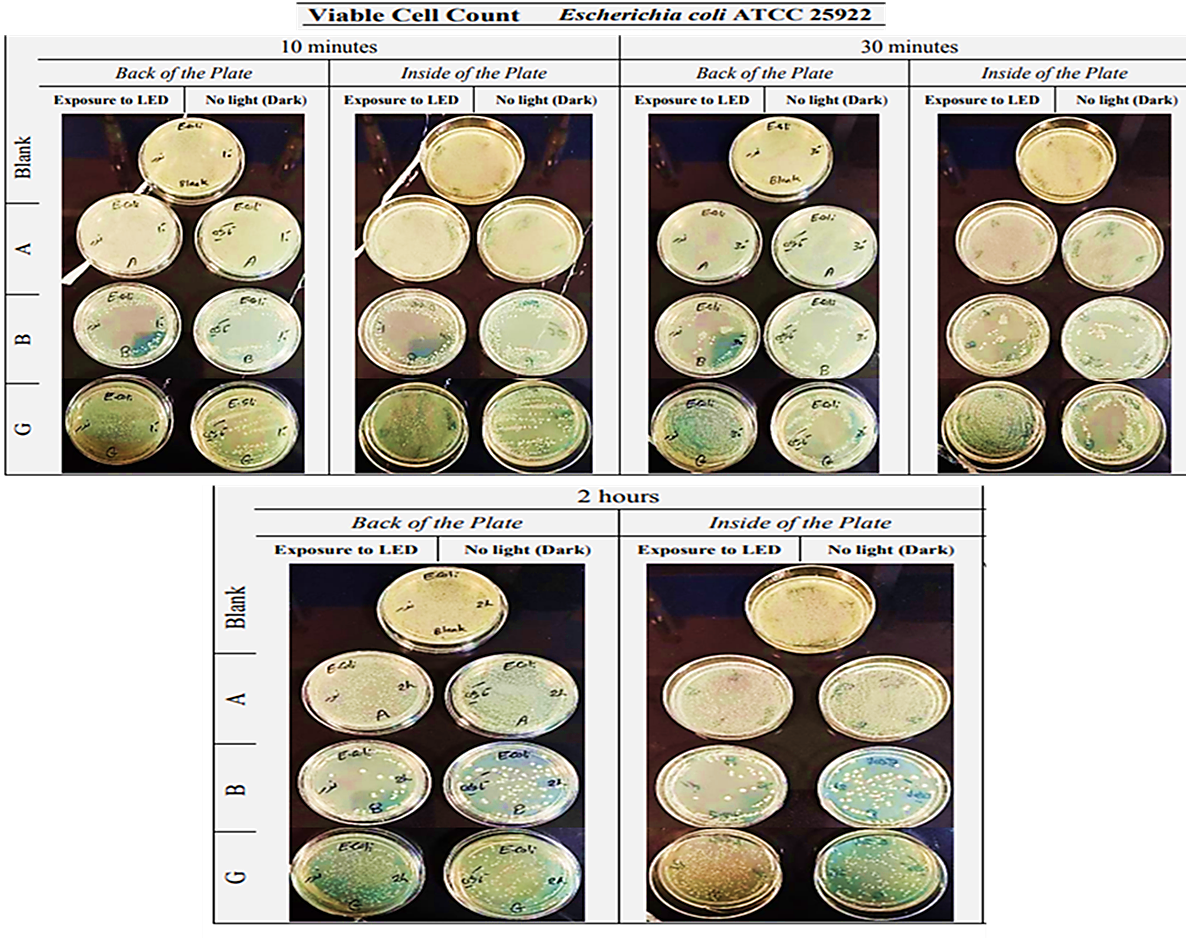


**Figure** **S5.** Photographs of CFU of *E. coli* in the presence and absence of nanocomposite with 2h LED light exposure and in dark conditions, after 24 h.

**
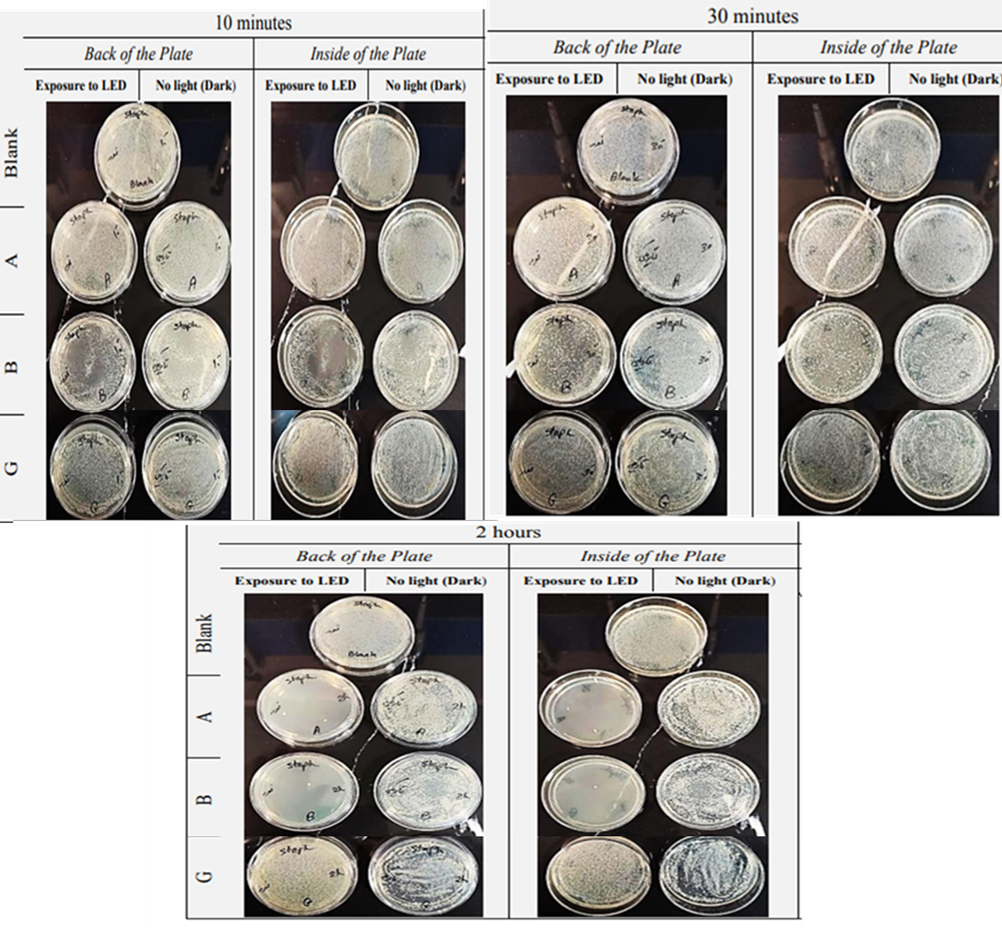
**

**Figure S6.** Photographs of CFU of *S. aureus* in the presence and absence of nanocomposite with 2h LED light exposure and in dark conditions, after 24 h.

**Table S2.** The minimum inhibitory concentration of *E. coli* in the presence of nanocomposite with LED light and in dark.

| Type *E. coli* (gram-negative bacteria) | | | |
| --- | --- | --- | --- |
| **ZnTPP/Au/AgCl-NPs** | **ZnTPP/Ag/AgCl/Cu-NPs** | **ZnTPP/Ag-NPs** | **Sample** |
| 78.1 | 9.7 | 39.0 | **MIC (ppm) (μg/ml)**  **Item (No light (Dark)** |
| 9.7 | <9.7 | 19.5 | **MIC (ppm) (μg/ml)**  **2 h Exposure to LED Lamp** |

**Table S3.** The minimum inhibitory concentration of *S. aureus* in the presence of nanocomposite with LED light and in dark.

| Type *S. aureus* (gram-positive bacteria) | | | |
| --- | --- | --- | --- |
| **ZnTPP/Au/AgCl-NPs** | **ZnTPP/Ag/AgCl/Cu-NPs** | **ZnTPP/Ag-NPs** | **Sample** |
| 78.1 | 39.0 | 156.2 | **MIC (ppm) (μg/ml)**  **Item (No light (Dark)** |
| 156.2 | <9.7 | 19.5 | **MIC (ppm) (μg/ml)**  **Exposure to LED Lamp** |

**Table S4.** The minimum bactericidal concentration of *S. aureus* in the presence of nanocomposite with LED light and in dark.

| Type *S. aureus (gram-positive bacteria)* | | | |
| --- | --- | --- | --- |
| **ZnTPP/Au/AgCl-NPs** | **ZnTPP/Ag/AgCl/Cu-NPs** | **ZnTPP/Ag-NPs** | **Sample** |
| 156.2 | 78.1 | 312.5 | **MBC (ppm) (μg/ml)**  **Item (No light (Dark)** |
| 312.5 | 9.7 | 39.0 | **MBC (ppm) (μg/ml)**  **Exposure to LED Lamp** |

| Type *E. coli* (gram-negative bacteria) | | | |
| --- | --- | --- | --- |
| **ZnTPP/Au/AgCl-NPs** | **ZnTPP/Ag/AgCl/Cu-NPs** | **ZnTPP/Ag-NPs** | **Sample** |
| 156.2 | 19.5 | 78.1 | **MBC (ppm) (μg/ml)**  **Item (No light (Dark)** |
| 19.5 | 9.7 | 39.0 | **MBC (ppm) (μg/ml)**  **Exposure to LED Lamp** |

**Table S5.** The minimum bactericidal concentration of *E. coli* in the presence of nanocomposite with LED light and in dark.

**Table S6**. Colony-forming unit and Reduction Percentage of *S. aureus* in the presence of nanocomposite in dark.

| Type *S. aureus (gram-positive bacteria)*  in the presence of nanocomposite in dark | | | |
| --- | --- | --- | --- |
| **ZnTPP/Au/AgCl-NPs** | **ZnTPP/Ag/AgCl/Cu-NPs** | **ZnTPP/Ag-NPs** | **Sample** |
| >9.5 × 10^5^ | >9.5 × 10^5^ | >9.5 × 10^5^ | \| CFU \| 10 min \| \| --- \| --- \| \| Reduction Percentage \| |
| <5 | <5 | <5 |  |
| 7.5 × 10^5^ | 9.5 × 10^5^ | 9.5 × 10^5^ | \| CFU \| 30 min \| \| --- \| --- \| \| Reduction Percentage \| |
| 15 | 10 | 5 |  |
| 3.5 × 10^5^ | 7 × 10^5^ | 8 × 10^5^ | \| CFU \| 2 h \| \| --- \| --- \| \| Reduction Percentage \| |
| 65 | 30 | 20 |  |

**Table S7**. Colony-forming unit and Reduction Percentage of *E. coli* in the presence of nanocomposites in dark.

| Type *E. coli* (gram-negative bacteria)  in the presence of nanocomposite in dark | | | |
| --- | --- | --- | --- |
| **ZnTPP-Au/AgCl-NPs** | **ZnTPP-Ag/AgCl/Cu-NPs** | **ZnTPP-Ag-NPs** | **Sample** |
| 1 × 10^5^ | 1 × 10^5^ | >9.5 × 10^5^ | \| CFU \| 10 min \| \| --- \| --- \| \| Reduction Percentage \| |
| 90 | 90 | <5 |  |
| 5 × 10^4^ | 5 × 10^4^ | >9.5 × 10^5^ | \| CFU \| 30 min \| \| --- \| --- \| \| Reduction Percentage \| |
| 90 | 95 | <5 |  |
| 1 × 10^3^ | <1 × 10^2^ | 9 × 10^5^ | \| CFU \| 2 h \| \| --- \| --- \| \| Reduction Percentage \| |
| >99.99 | >99.99 | 10 |  |

**Table S8.** Colony-forming unit and Reduction Percentage of *S. aureus* in the presence of nanocomposite in light condition.

| Type *S. aureus (gram-positive bacteria)*  In the presence of nanocomposite with LED light after 24h. | | | |
| --- | --- | --- | --- |
| **ZnTPP/Au/AgCl-NPs** | **ZnTPP/Ag/AgCl/Cu -NPs** | **ZnTPP/Ag-NPs** | **Sample** |
| 9.5 × 10^5^ | 6 × 10^5^ | 9.5 × 10^5^ | \| CFU \| 10 min \| \| --- \| --- \| \| Reduction Percentage \| |
| 0 | 40 | 5 |  |
| 7.5 × 10^5^ | 2 × 10^5^ | 9 × 10^5^ | \| CFU \| 30 min \| \| --- \| --- \| \| Reduction Percentage \| |
| 15 | 80 | 10 |  |
| 7.5 × 10^5^ | <1 × 10^1^ | <1 × 10^1^ | \| CFU \| 2 h \| \| --- \| --- \| \| Reduction Percentage \| |
| 25 | >99.99 | >99.99 |  |

| **ZnTPP/Au/AgCl-NPs** | **ZnTPP/Ag/AgCl/Cu-NPs** | **ZnTPP/Ag-NPs** | **Sample** |
| --- | --- | --- | --- |
| 7 × 10^5^ | 1 × 10^5^ | >9.5×10^5^ | \| CFU \| 10 min \| \| --- \| --- \| \| Reduction Percentage \| |
| 30 | 90 | <5 |  |
| 4 × 10^5^ | 5 × 10^4^ | >9.5 × 10^5^ | \| CFU \| 30 min \| \| --- \| --- \| \| Reduction Percentage \| |
| 60 | 95 | <5 |  |
| 1 × 10^5^ | <1 × 10^2^ | 6 × 10^5^ | \| CFU \| 2 h \| \| --- \| --- \| \| Reduction Percentage \| |
| 90 | >99.99 | 40 |  |

| Type *E. coli* (gram-negative bacteria)  In the presence of nanocomposite with LED light after 24h. |
| --- |

**Table S9.** Colony-forming unit and Reduction Percentage of *E. coli* in the presence of nanocomposite in light condition.

**Reference**

1. Tehrani Nejad, S., Rahimi, R., Rabbani, M., Rostamnia, S. Zn (II)-porphyrin-based photochemically green synthesis of novel ZnTPP/Cu nanocomposites with antibacterial activities and cytotoxic features against breast cancer cells. *Sci. Rep*. (2022) 12:17121
